# A systematic review and meta-analyses of the relationships between active outdoor play and 24-hour movement behaviors

**DOI:** 10.1016/j.jshs.2025.101115

**Published:** 2025-12-29

**Authors:** Maeghan E. James, Louise de Lannoy, Olivia Lopes, Avril Johnstone, Eun-Young Lee, Peter Bakalár, Javier Brazo-Sayavera, Taru Manyanga, Leigh M. Vanderloo, Erin Wentzell, Lisa M. Barnett, Peter Bentsen, Valerie Carson, Scott Duncan, Ryan Fahey, Shawnda A. Morrison, Lærke Mygind, Alessandra Prioreschi, Suryeon Ryu, Lindsey Sikora, Patricia Tucker, Lucy-Joy Wachira, Mark S. Tremblay

**Affiliations:** a Healthy Active Living and Obesity Research Group, Children’s Hospital of Eastern Ontario Research Institute, Ottawa, ON K1H 8L1, Canada; b Faculty of Medicine, University of Ottawa, Ottawa, ON K1H 8M5, Canada; c Outdoor Play Canada, Ottawa, ON K2K 2Y1, Canada; d MRC/CSO Social and Public Health Sciences Unit, University of Glasgow, Glasgow G12 8TB, UK; e School of Kinesiology & Health Studies, Queen’s University, Kingston, ON K7L 3N6, Canada; f Faculty of Sports, University of Prešov, Prešov 08001, Slovakia; g Faculty of Physical Culture, Palacký University Olomouc, Olomouc 771 47, Czech Republic; h Department of Sports and Computer Science, Universidad Pablo de Olavide, Sevilla 41013, Spain; i Division of Medical Sciences, University of Northern British Columbia, Prince George, BC V2N 4Z9, Canada; j Research & Evaluation, ParticipACTION, Toronto, ON M5G 2C8, Canada; k School of Occupational Therapy, University of Western Ontario, London, ON N6A 3K7, Canada; l Department of Health, Human Function, and Rehabilitation Sciences, The George Washington University, Washington, DC 20037, USA; m Institute for Physical Activity and Nutrition, School of Health and Social Development, Deakin University, Geelong, VIC 3220, Australia; n Center for Clinical Research and Prevention, University Hospital Copenhagen – Bispebjerg and Frederiksberg, Capital Region 2400, Denmark; o Department of Geosciences and Natural Resource Management, University of Copenhagen, Copenhagen 1958, Denmark; p Faculty of Kinesiology, Sport, and Recreation, University of Alberta, Edmonton, AB T6G 2H9, Canada; q School of Sport and Recreation, Auckland University of Technology, Auckland 1142, New Zealand; r Physical and Health Education Canada, Ottawa, ON K1H 7X7, Canada; s Yong Loo Lin School of Medicine, National University of Singapore, Singapore 117597, Singapore; t SAMRC Developmental Pathways for Health Research Unit, Department of Pediatrics, Faculty of Health Science, School of Clinical Medicine, University of the Witwatersrand, Johannesburg 2050, South Africa; u Department of Kinesiology, Recreation, and Sport Studies, University of Tennessee, Knoxville, TN 37996, USA; v Health Sciences Library, University of Ottawa, Ottawa, ON K1H 8M5, Canada; w Children’s Health Research Institute, London, ON N6C 2V5, Canada; x Department of Physical Education, Exercise and Sport Science, Kenyatta University, Nairobi 00100, Kenya

**Keywords:** Children, Physical activity, Sedentary behavior, Sleep, Public health

## Abstract

**Background:**

Few individuals meet the 24-h movement guidelines for physical activity (PA), sedentary behavior, and sleep. Active outdoor play (AOP) may support healthier movement patterns, though its role is not yet established. The objective of this study was to synthesize evidence on associations between AOP and movement behaviors.

**Methods:**

A systematic review and meta-analyses were conducted using 5 databases from inception to September 2025. Studies examining AOP and movement behaviors were included with no restriction on age, ability, or geographic location. Screening and data extraction were completed in duplicate. Narrative syntheses, random-effects meta-analyses, and The Grading of Recommendations Assessment, Development, and Evaluation assessments were conducted.

**Results:**

Of 28,092 records, 61 studies met inclusion criteria, spanning 25 countries with participants aged 1.6–15.5 years. Most were cross-sectional (*n* = 46), with some longitudinal (*n* = 8), quasi-experimental (*n* = 5), and randomized trials (*n* = 3). AOP was consistently associated with greater PA, especially moderate-to-vigorous PA; meta-analysis showed a moderate positive correlation with high heterogeneity (*r* = 0.60, 95% confidence interval (95%CI): 0.34–0.78, *p* = 0.0004; *I*^2^ = 99.2%). AOP was also linked to less sedentary behavior: children were sedentary 38% of the time outdoors *vs.* 67% indoors. Meta-analyses showed a small, significant negative correlation with sedentary time (*r* = –0.05, 95%CI: –0.07 to –0.02, *p* < 0.0043; *I*^2^ = 7.0%) and a small, non-significant correlation with screen time (*r* = –0.19, 95%CI: –0.38 to 0.02, *p* = 0.0638; *I*^2^ = 98.9%). Evidence on sleep was mixed, with no adverse associations found. Certainty of evidence was low to very low.

**Conclusion:**

AOP may support healthier movement behaviors in children and youth. More longitudinal and intervention research is needed to confirm causality and guide policy and practice.

## Highlights

• Active outdoor play is positively associated with physical activity, particularly moderate-to-vigorous intensity activity among children and youth.
• Children are more active and less sedentary outdoors than indoors, and active outdoor play is linked to lower screen time.
• Active outdoor play interventions may increase physical activity and reduce sedentary behavior.
• Evidence suggests active outdoor play supports healthier movement behaviors, though more longitudinal and intervention research is needed across the lifespan.

## Graphical abstract

**Figure:**
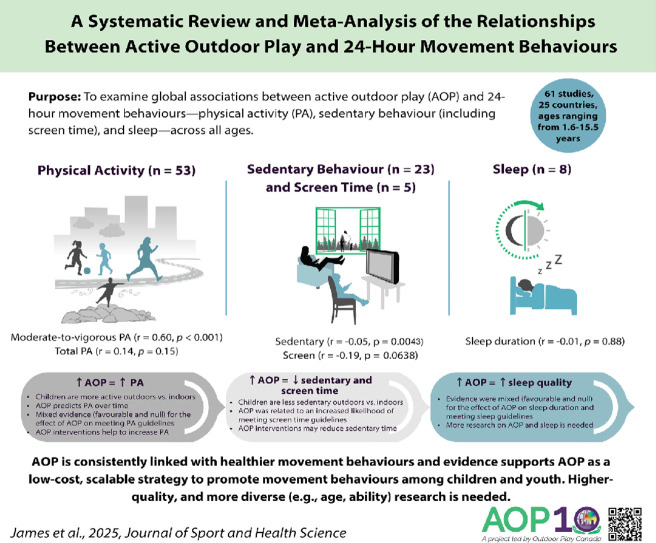

## 1 Introduction

An optimal balance of physical activity (PA), sedentary time, and sleep are essential components of a healthy lifestyle for people of all ages.^1^^,^^2^ Recognizing the importance of a healthy balance of these movement behaviors throughout the day, 24-h movement behavior guidelines (hereafter: movement behavior guidelines) were developed in Canada to provide recommendations on the amounts of PA, sedentary behavior, and sleep that should be incorporated into a 24-h day across different age groups (Table 1).^3^, ^4^, ^5^ In response, several countries have adopted this “24-hour approach” to movement behaviors.^6^, ^7^, ^8^ Adhering to these guidelines contributes to better physical, mental, and cognitive health outcomes, including a reduced risk of chronic disease, improved emotional well-being, and enhanced daily functioning.^2^^,^^4^^,^^9^^,^^10^ Despite the known benefits of engaging in recommended movement behaviors, the majority of individuals fail to meet these recommendations, with a recent systematic review reporting low adherence rates among children, youth, and adults alike.^11^ Thus, concerted efforts are needed to determine and implement effective public health strategies globally to improve movement behavior guideline adherence for all.

**Table 1:** Summary of Canadian movement behavior guidelines across age groups.

Age group	PA	Sedentary behavior	Sleep
**Infants (<1 year)**	Physically active several times per day At least 30 min of tummy time per day for those not yet mobile	≤1 h at a time of being restrained Screen time not recommended	0–3 months: 14–17 h per day 4–11 months: 12–16 h per day (including naps)
**Toddlers (1–2 years)**	180 min per day of PA at any intensity	≤1 h at a time being restrained or sedentary Screen time not recommended for those under 2 years ≤1 h of screen time for those aged 2 years	11–14 h (including naps)
**Preschoolers (3–4 years)**	180 min per day of PA at any intensity At least 60 min of MVPA per day	≤1 h at a time being restrained or sedentary ≤1 h of screen time	10–13 h (may include naps)
**Children (5–13 years)**	60 min of MVPA per day Several hours of LPA	≤2 h of screen time per day	9–11 h per night
**Youth (14–17 years)**	60 min of MVPA per day Several hours of LPA	≤2 h of screen time per day	8–10 h per night
**Adults (18–64 years)**	150 min of MVPA per week	Limit sedentary time to <8 h ≤3 h of screen time	7–9 h per night
**Older adults (65+ years)**	150 min of MVPA per week	Limit sedentary time to <8 h ≤3 h of screen time	7–8 h per night

Note: This table presents a high-level summary of movement behavior guidelines across age groups. For a complete description of guidelines visit: https://csepguidelines.ca/.

Abbreviations: LPA = light physical activity; MVPA = moderate-to-vigorous physical activity; PA = physical activity.

Active outdoor play (AOP) is increasingly recognized as an effective vehicle to enhance health and well-being.^12^ Specifically, engaging in AOP can increase PA, reduce sedentary behaviors, and improve sleep.^13^ Some studies also report that spending time outdoors leads to better health outcomes in adult and older adult populations.^14^, ^15^, ^16^ AOP, as defined by Lee and colleagues,^17^ is the “voluntary engagement in activity that takes place outdoors, involves PA of any intensity, is fun and/or rewarding and usually driven by intrinsic motivation”. In children and youth, AOP often consists of unstructured activities, such as playing on a playground, riding a bike, or swimming. In adults, while the term “play” may not always be used directly, AOP can include any PA that a person engages in willingly for the sake of enjoyment (e.g., hiking, kayaking) and without the structured rules that apply for sport. In 2015, a systematic review by Gray et al.^18^ (based on 28 studies) found that AOP was related to increased PA and reduced sedentary behaviors in children (aged 3–12 years). That review served as a foundational piece of evidence used to advocate for a shift in AOP perspectives, urging parents, educators, and policymakers in Canada to prioritize AOP as a fundamental component of healthy childhood development. Since the 2015 Position Statement on Active Outdoor Play^19^ was developed, the importance of AOP has gained recognition, not only in academic literature but also within health policy and clinical practice.^20^, ^21^, ^22^ For instance, the Council of Chief Medical Officers of Health in Canada and the Canadian Pediatric Society have emphasized the importance of outdoor play for children’s health.^12^^,^^21^ In fact, some medical professionals are now prescribing outdoor play as a therapeutic strategy to improve health outcomes.^23^ This growing integration of outdoor play into clinical guidance underscores its emerging role in public health strategies and signals strong potential for even greater emphasis in future global public health initiatives.

Since the seminal review by Gray and colleagues^18^ and the release of the 2015 Position Statement on Active Outdoor Play,^19^ AOP research has expanded significantly, with a tenfold increase in research on AOP in Canada alone.^24^^,^^25^ While Gray et al.’s^18^ review provided important information on how AOP may promote PA and reduce sedentary behavior, there are some key areas to be further developed. First, the review was conducted *prior to* the first Position Statement on Active Outdoor Play in 2015 and, thus, did not have access to the wider expanse of studies that followed the publication of that statement. Second, the review was conducted *prior to* the adoption of the movement behavior guidelines in 2016. As such, the review did not consider the full spectrum of 24-h movement behaviors (i.e., it did not include sleep and screen time behaviors). Lastly, their search strategy was limited to children and youth only, leaving out the potential of AOP among older age groups. Given the growing recognition of the interconnectedness of movement behaviors and the explosive increase in AOP research since 2015, there is a need for a 10-year follow-up to the original review. Expanding the research scope criteria to include sleep and screen time and expanding to adult populations provides a more comprehensive understanding of the potential role of AOP in overall daily movement behaviors for all.

This systematic review and meta-analyses aimed to determine the relationship between AOP and the 24-h movement behaviors—specifically, PA, sedentary behavior (including screen time) and sleep—among children, adolescents, and adults, globally. This review serves as an update to the previous systematic review by Gray et al.^18^ while expanding the search to include sleep, screen time, and individuals of all ages.

## 2 Methods

### 2.1 Study design

The systematic review and meta-analyses were conducted in accordance with the Preferred Reporting Items for Systematic Reviews and Meta-Analyses (PRISMA) guidelines^26^ (Supplementary Table 1) and registered on PROSPERO (Protocol #CRD42024517145). This study was part of a larger, global project updating the 2015 Position Statement on Active Outdoor Play in recognition of its 10-year anniversary (hereafter referred to as the “AOP10 project”).^27^ This review was conducted in line with the evidence-informed framework developed by the international leadership team for the AOP10 project to address the theme of movement behaviors and its relationship to AOP.^28^

### 2.2 Eligibility criteria

Studies were eligible to be included in this review if they examined the relationship between AOP and engagement in movement behaviors (i.e., PA, sedentary behavior, screen time, and sleep) across all age groups and abilities. These eligibility criteria were determined collaboratively by the full authorship team and leadership group for the AOP10 project using the Population, Intervention/Exposure, Comparators, Outcomes, Study design (PICOS) framework. Criteria for each PICOS indicator are described below.

#### 2.2.1 Population

Studies were eligible if they focused on children, youth, and/or adults of any age and ability, from any country. To capture international representation, studies published in any of the 6 official United Nations languages (Arabic, Chinese, English, French, Russian, and Spanish) were eligible.

#### 2.2.2 Intervention/Exposure

The primary exposure of interest was AOP, defined earlier.^17^ Studies were only included if the exposure variable specifically indicated a frequency or duration of time playing or being active outdoors. To identify literature examining a range of outdoor play activities, any form of outdoor PA performed for leisure purposes was included, regardless of whether the term “play” was explicitly used. This review excluded studies focusing on organized sport (e.g., youth soccer program), occupational outdoor activities (e.g., outdoor labor), or structured health interventions (e.g., outdoor exercise prescribed for weight loss). For adults, professional sport was excluded on the basis that it is typically performed as an occupation or for monetary gain. This review did not include proximal measures of outdoor play such as distance to or frequency of visits to an outdoor space. Time spent outdoors was excluded if no mention of play or leisure activity were stipulated. This review considered potential bi-directional relationships between 24-h movement behaviors and AOP. All outdoor settings were considered, including (but not limited to) yards, driveways, parks, playgrounds, childcare and school grounds, trails, and walking paths. AOP could occur at any time of day, day of the week, climate, season, or weather conditions. For intervention studies, we included those in which outdoor play was supported through environmental or policy modifications (e.g., closing streets to traffic, increasing outdoor time in schools) rather than through direct instruction or assignment to engage in outdoor play. Studies were excluded if the intervention involved prescribing or directing participants’ engagement in outdoor play (e.g., being assigned to walk outdoors 3 times per week).

#### 2.2.3 Comparators

Comparators included varying levels of AOP exposure as well as non-AOP control groups, such as indoor active play or indoor and outdoor non-active play.

#### 2.2.4 Outcomes

The primary outcome of interest was the direction and strength of the relationship between AOP and movement behaviors. Quantitative studies assessing the relationship between AOP and PA, sedentary behavior, screen time, sleep, or any combination thereof were included in this review. There were no restrictions on the type of measurement tools used. Studies employing self-report, proxy-report, device-based, or observational methods were all considered.

#### 2.2.5 Study design

This review included randomized controlled trials (RCTs) and non-RCTs, such as cross-sectional studies, retrospective and prospective cohort studies, case-control studies, and longitudinal studies.

### 2.3 Search strategy

A comprehensive search strategy was developed and piloted in collaboration with a health sciences librarian (LS). The literature search was conducted by the librarian using the following databases: MEDLINE and MEDLINE in Process (Ovid), Embase Classic+Embase (Ovid), CINAHL (EBSCOHost), SPORTDiscus (EBSCOHost), and Scopus. The search strategy was initially developed in Medline and then adapted for the other databases as appropriate (Supplementary Table 2). To ensure rigor, the Medline search was peer-reviewed by another librarian using the Peer Review of Electronic Search Strategies (PRESS) tool. The original search was conducted on June 20, 2024 and updated on September 25, 2025 to capture newly published studies. Publication filters were applied to identify RCTs and observational studies using Canadian Agency for Drugs and Technologies in Health (CADTH) search filters.

### 2.4 Study selection process

Study selection was conducted using Covidence and following the best practice guidelines for large-scale systematic reviews developed by Polanin et al.,^29^ including the use of clear inclusion/exclusion criteria, dual independent screening, and the documentation of decision rules and reviewer consensus processes. Independent double-screening was employed at 2 levels. At Level 1 (title and abstract screening), 10 reviewers (MEJ, OL, LJW, LdL, PBe, LMV, AJ, PT, VC, and TM) assessed articles for potential inclusion, with uncertain cases advancing to full-text review. Inter-rater reliability demonstrated high agreement, with proportionate agreement ranging from 0.85 to 1.00 and Cohen’s *kappa* values ranging from 0.00 to 0.67 across reviewer pairs. Disagreements were reconciled by a third author (MEJ, LdL, or OL). At Level 2 (full-text screening), 7 reviewers (MEJ, PBa, LdL, AJ, TM, Brianna Nasrallah, and SD) independently assessed full-text articles using pre-defined inclusion/exclusion criteria. Discrepancies were resolved through consensus discussions, with a 3rd reviewer (MEJ, LdL, or OL) making final decisions if needed.

### 2.5 Data extraction

Data were extracted by 6 team members (MEJ, LB, AJ, JBS, LM, and Anujah Thankarajah) using Covidence. Extracted information included bibliographic details (e.g., year of publication, author name) and data corresponding to each of the PICOS variables, including relevant descriptive statistics (e.g., means, standard deviations, counts, and percentages) and inferential statistics (e.g., test statistics, regression coefficients, *p* values, confidence intervals, effect sizes) where reported. Each study was assigned to two reviewers, with one extracting the data and the other verifying it. Discrepancies were resolved through discussion, and if consensus was not reached, a 3rd reviewer was consulted. Any remaining disagreements were resolved by the primary investigator (MEJ).

### 2.6 Quality appraisal

The Joanna Briggs Institute’s critical appraisal tools (https://jbi.global/critical-appraisal-tools) were used to assess the risk of bias in the included studies. Bias was judged as yes, no, unclear, or not applicable across domains specific to each study design. If an item was marked as “not applicable”, it was excluded from the final percentage calculation. Risk of bias assessment was conducted independently by pairs of reviewers (JBS, AJ, LM, and Anujah Thankarajah) with discrepancies resolved through discussion.

### 2.7 Certainty of evidence

The Grading of Recommendations Assessment, Development, and Evaluation (GRADE) approach was used to assess the certainty of evidence for each outcome,^30^ separated by study design.^31^ Certainty of evidence was rated as high, moderate, low, or very low based on 5 domains: risk of bias, inconsistency, indirectness, imprecision, and publication bias. Initially, RCTs were rated as high-certainty and non-randomized designs (i.e., cross-sectional, longitudinal, quasi-experimental) as low-certainty, with downgrades or upgrades applied as appropriate. Two reviewers (RF and EW) independently rated the certainty of evidence, and discrepancies were resolved through discussion and consultation with the primary investigator (MEJ).

### 2.8 Data synthesis and statistical analysis

Quantitative meta-analyses were undertaken when at least 2 effect sizes from 2 different studies were available and considered comparable for a given relationship.^32^ We considered studies comparable if they used similar study designs, measured a duration of AOP as the exposure, and assessed a duration-based movement behavior outcome. To maximize the data included in the meta-analyses, we accepted both parent-report and accelerometry-based measures of movement behaviors, as both are widely used, valid, and reliable assessment methods.^33^ When a study reported on more than one outcome related to the movement behavior of interest (e.g., different measures of PA) or reported on associations across different sub-groups (e.g., by sex/gender groups), each was extracted and included as a separate data point in the meta-analyses. This approach allowed us to capture the full range of relevant evidence, though it introduced a degree of statistical dependence between some data points from the same study. The primary effect size metric was the Pearson correlation coefficient (*r*). Correlation coefficients reported in the original studies were used directly. Where only regression coefficients were provided, the unadjusted regression coefficient was converted to a Pearson correlation coefficient using the esc B() function from the esc package in R.^34^ Odds ratios were converted to Pearson correlation coefficients using the oddsratio_to_r() function from the effect size R package,^35^ incorporating group sample sizes where available to improve the accuracy of the transformation. To ensure consistency in the direction of effects, all effect sizes were standardized such that positive values for PA and sleep indicate that increased AOP is associated with increased PA and sleep, and negative values for sedentary behavior and screen time indicate that increased AOP is associated with decreased sedentary behavior and screen time. Where necessary, effect sizes were reverse-coded. All meta-analyses were conducted using a random-effects model with restricted maximum likelihood estimation, implemented via the metacor() function in the meta package in R. The metacor() function^36^ internally transforms *r* values to Fisher’s *z*-scores to stabilize variance and normalize the distribution for meta-analytic calculations. Pooled estimates were then back-transformed to *r* values for interpretability. Correlation coefficients were interpreted as negligible (0.00–0.09), weak (0.10–0.39), moderate (0.40–0.69), strong (0.70–0.89), or very strong (0.90–1.00).^37^ Between-study heterogeneity was assessed using the *I*^2^.^38^ Confidence intervals were used to assess precision of the results and infer adequacy of statistical power. We used the width and boundaries of the confidence intervals around pooled effect estimates to assess the precision of the results and infer the adequacy of statistical power. Confidence intervals that spanned both negligible (*r*: 0.00–0.10) and meaningful (*r* > 0.10) effect sizes were interpreted as indicating limited statistical power and imprecision, making it difficult to draw definitive conclusions about the strength of the association. Forest plots were used to display the results from the meta-analyses. Subgroup analyses were initially considered to examine results by socio-demographic factors (e.g., socio-economic status, disability status); however, these data were not available in the included studies.

In addition to meta-analyses, a narrative synthesis was conducted to summarize the associations between AOP and each outcome category: (a) PA, (b) sedentary behavior (including screen time), and (c) sleep across all included studies. For each included study, the association between AOP and the movement behavior(s) of interest was recorded as favorable, unfavorable, null, or mixed findings based on the significance and direction of the effect. Direction of effect followed the same pattern previously discussed for the meta-analyses. All statistical analyses in which AOP was entered as either the independent or dependent variable were considered. In a single paper, multiple models were reported so effect sizes from fully adjusted models were prioritized; otherwise, unadjusted estimates were used. If a study reported multiple effect sizes, such as when it examined more than one movement behavior (e.g., both PA and sleep) or assessed the same behavior using different measures (e.g., self-report and accelerometry), each effect size was included and treated as a separate data point in the synthesis. Only findings with statistical significance (*p* < 0.05) were considered indicative of a meaningful association. The consistency of the evidence was categorized based on the proportion of observations supporting the hypothesized association: 0%–33% (*no evidence*), 34%–59% (*inconsistent evidence*), and 60%–100% (*consistent evidence*).

## 3 Results

### 3.1 Description of studies

A total of 28,092 studies were identified through database searches. After duplicates were removed, 22,226 studies were screened based on their titles and abstracts. Of the 488 studies screened for full text, 61 papers (62 separate studies) met the inclusion criteria and were included in the review. A summary of study characteristics is presented in Supplementary Table 3.

**Fig. 1:**
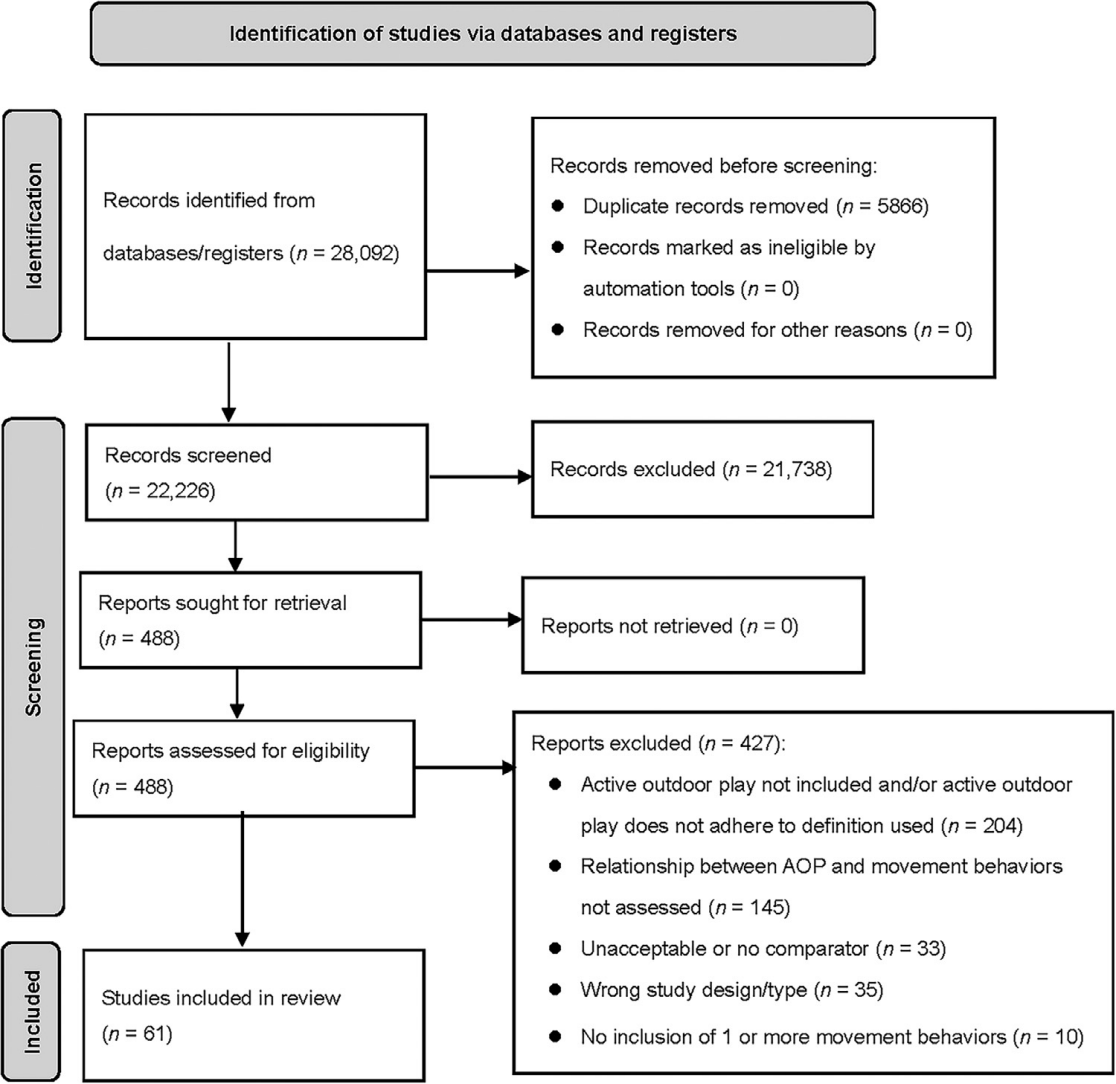
The preferred reporting items for systematic reviews and meta-analyses flow diagram. AOP = active outdoor play.

The included studies (Fig. 1) investigated a direct relationship between AOP and PA (*n* = 53), sedentary behavior (*n* = 23), screen time (*n* = 5), and sleep (*n* = 8). Most study designs were cross-sectional (*n* = 46), followed by longitudinal (*n* = 8), quasi-experimental (*n* = 5), and RCT (*n* = 3) designs. Studies were conducted across 25 countries, most frequently the USA (*n* = 19), Canada (*n* = 8), the UK (*n* = 5), and Australia (*n* = 5). Participant ages ranged from infancy to adolescence (1.6–15.5 years), with the majority of studies (*n* = 55) focusing on early- to middle-childhood (ages 2–12 years). Although this review intended to include people of all ages, no studies involving adults met the inclusion criteria for this review, and possible reasons for this observation are addressed subsequently in the discussion. No studies reported disaggregated data on equity-deserving groups (e.g., racialized, low income, sexual identity, people with disability, elderly); however, 1 study was identified that focused on children with disabilities.^39^

### 3.2 Risk of bias and certainty of evidence

#### 3.2.1 Risk of bias

Common sources of bias varied by study design. Cross-sectional studies generally provided clear inclusion criteria and appropriate statistical analyses but many failed to use measurement tools of AOP with evidence of validity and reliability (43%), indicating risk of measurement bias. Longitudinal studies consistently addressed confounding factors (100%) and used appropriate analyses (100%) but often failed to adequately report or address incomplete follow-up (71%). Quasi-experimental studies clearly established causal direction and measured outcomes consistently but only half adequately handled statistical analysis and group comparability, and just a few addressed follow-up bias (25%). While RCTs (*n* = 3) met some methodological standards, they did not report allocation concealment or blinding of participants, personnel, or assessors, posing risks of performance and detection bias. However, it should be acknowledged that the Joanna Briggs Institute’s critical appraisal tools were developed primarily for clinical research, and some criteria (e.g., participant blinding) may not be feasible or fully applicable in behavioral intervention studies such as those involving AOP. As such, these quality assessments should be interpreted with caution within this context. A complete summary of risk of bias assessments by study design is available in Supplementary Table 4.

#### 3.2.2 Certainty of evidence

The certainty of evidence ranged from low to very low, primarily due to the observational nature of the included studies. In several cases, downgrades were applied for risk of bias, inconsistency, or imprecision, particularly when findings were mixed, sample sizes were small, or follow-up data were incomplete. Several studies used instruments lacking validity evidence to assess AOP, reflecting a broader limitation within the field; these were not considered serious enough to warrant downgrading. A detailed GRADE assessment, including domain-level justifications, is provided in Supplementary Table 5.

### 3.3 Data synthesis

Meta-analyses and narrative syntheses were performed for all movement behavior outcomes individually, across all study designs. Meta-analyses are detailed for each movement behavior individually, followed by their narrative synthesis, and further summarized in Table 2.

**Table 2:** Relationships between active outdoor play and the 24-h movement behaviors.

Total sample (number of studies)	Study design	Summary of findings	Certainty of evidence^a^
**PA**			
41,249 (38)	Cross-sectional	**Any intensity PA (*n*** **=** **15 associations)** 11/15 (73.3%) studies showed favorable associations,^39^^,^^47^^,^^52^^,^^54^^,^^60^^,^^75^, ^76^, ^77^^,^^83^^,^^86^^,^^87^^,^^90^ 1/15 showed mixed (favorable and null) associations,^65^ 2/15 showed no association^61^^,^^91^ **Light PA (*n*** **=** **6 associations)** 5/6 (83.3%) studies showed favorable associations,^43^^,^^63^^,^^79^^,^^82^^,^^87^ 1/6 studies showed no association^61^ **Moderate-to-vigorous PA (*n*** **=** **24 associations)** 18/24 (75%) studies showed favorable associations,^40^^,^^42^^,^^43^^,^^48^^,^^50^^,^^56^, ^57^, ^58^^,^^62^^,^^67^^,^^68^^,^^74^^,^^79^^,^^80^^,^^82^^,^^84^^,^^87^^,^^90^ 2/24 showed mixed associations,^71^^,^^86^ 4/24 showed no association^61^^,^^63^^,^^75^^,^^76^ **Steps per day (*n*** **=** **3 associations)** 3/3 (100%) studies showed favorable associations^40^^,^^43^^,^^66^ **Meeting PA guidelines (*n*** **=** **6 associations)** 3/6 (50%) showed favorable associations,^72^, ^73^, ^74^ 3/6 showed no association^61^^,^^64^^,^^77^ **Overall (across all PA outcomes; *n*** **=** **53 associations)** 40/53 (75.5%) associations were favorable, 4/53 associations were mixed (favorable and null), 9/53 associations yielded null results	⊕○○○ LOW
14,087 (6)	Longitudinal	**Total PA (*n*** **=** **4 associations)** 3/4 (75%) studies showed favorable associations,^55^^,^^59^^,^^70^ 1/4 showed no association^78^ **Moderate-to-vigorous PA (*n*** **=** **2 associations)** 1/2 (50%) studies showed a favorable association,^89^ 1/2 studies showed no association^67^ **Overall (across all PA outcomes; *n*** **=** **6 associations)** 4/6 (66.7%) associations were favorable, 2/6 associations yielded null results	⊕⊕○○ LOW
1380 (8)	Outdoor play interventions – quasi experimental and randomized controlled trials	**Total PA (*n*** **=** **4 associations)** 3/4 (75%) studies showed no intervention effect,^41^^,^^44^^,^^88^ 1/4 studies showed mixed intervention effects (favorable and null)^45^ **Light PA (*n*** **=** **1 association)** 1/1 (100%) study showed no intervention effect^81^ **Moderate-to-vigorous PA (*n*** **=** **6 associations)** 1/6 (16.7%) studies showed a favorable intervention effect,^49^ 2/6 studies showed mixed intervention effects (favorable and null),^51^^,^^85^ 3/5 studies showed no intervention effect^41^^,^^81^^,^^88^ **Steps per day** 1/1 (100%) study showed a favorable intervention effect^49^ **Meeting guidelines** 2/2 (100%) studies showed no intervention effect^44^^,^^45^	Non-randomized trials: ⊕○○○ VERY LOW Randomized trials: ⊕○○○ VERY LOW
**Sedentary behavior**			
			
16,427 (18)	Cross-sectional	12/18 (66.7%) studies showed favorable associations with sedentary time,^40^^,^^42^^,^^58^^,^^63^^,^^67^^,^^74^^,^^75^^,^^79^^,^^80^^,^^82^^,^^86^^,^^110^ 1/18 studies showed mixed (favorable and null) associations,^65^ 5/18 studies showed no associations^57^^,^^61^^,^^68^^,^^91^^,^^92^	⊕⊕○○ LOW
392 (4)	Outdoor play interventions – quasi experimental and randomized controlled trials	1/4 (25%) interventions had favorable effects on sedentary behavior,^51^ 1/4 showed mixed effects (favorable and null),^85^ 2/4 showed no effect^44^^,^^81^	Non-randomized trials: ⊕○○○ VERY LOW Randomized trials: ⊕○○○ VERY LOW
**Screen time**			
11,937 (5)	Cross-sectional	**Screen time (*n*** **=** **2 associations)** 2/2 (100%) studies showed a favorable association^91^^,^^93^ **Meeting screen time guidelines (*n*** **=** **3 associations)** 2/3 (66.7%) showed favorable associations,^64^^,^^73^ 1/3 showed no association^61^ **Overall (across all screen time outcomes)** 4/5 (80%) showed favorable associations, 1/5 showed no association	⊕⊕○○ LOW
3442 (2)	Longitudinal	**Screen time** 1/2 (50%) showed mixed (favorable and null) effects^67^ and 1/2 showed mixed (favorable and unfavorable) effects^94^	⊕○○○ VERY LOW
**Sleep** 61,320 (8)	Cross-sectional	**Sleep quality** 2/2 (100%) studies showed a favorable association^95^^,^^98^ **Sleep quantity** 1/3 (33.3%) studies showed a favorable association,^96^ 1/3 studies showed mixed associations (favorable and null),^98^ 1/3 studies showed no association^69^ **Meeting sleep guidelines** 1/4 (25%) studies showed favorable associations,^97^ 1/4 studies showed mixed associations (favorable and null),^73^ 2/4 studies showed no association^53^^,^^64^ **Overall (across all sleep outcomes)** 4/9 (44.4%) studies showed favorable associations, 2/9 studies showed mixed associations (favorable and null), 3/9 studies showed no association	⊕○○○ VERY LOW

a For a complete GRADE analysis, including justifications for downgrading across each GRADE domain, see Supplementary Table 3. Abbreviations: GRADE = Grading of Recommendations Assessment, Development, and Evaluation; PA = physical activity.

#### 3.3.1 PA

A total of 53 studies^39^, ^40^, ^41^, ^42^, ^43^, ^44^, ^45^, ^46^, ^47^, ^48^, ^49^, ^50^, ^51^, ^52^, ^53^, ^54^, ^55^, ^56^, ^57^, ^58^, ^59^, ^60^, ^61^, ^62^, ^63^, ^64^, ^65^, ^66^, ^67^, ^68^, ^69^, ^70^, ^71^, ^72^, ^73^, ^74^, ^75^, ^76^, ^77^, ^78^, ^79^, ^80^, ^81^, ^82^, ^83^, ^84^, ^85^, ^86^, ^87^, ^88^, ^89^, ^90^, ^91^ examined the relationship between AOP and PA (Table 2). PA outcomes included those defined as: light PA (*n* = 6), moderate-to-vigorous PA (MVPA; *n* = 24), any PA (no intensity specified, *n* = 14), steps per day (*n* = 3), and meeting country-specific PA guidelines (*n* = 6).

##### 3.3.1.1 Meta-analyses

**Fig. 2:**
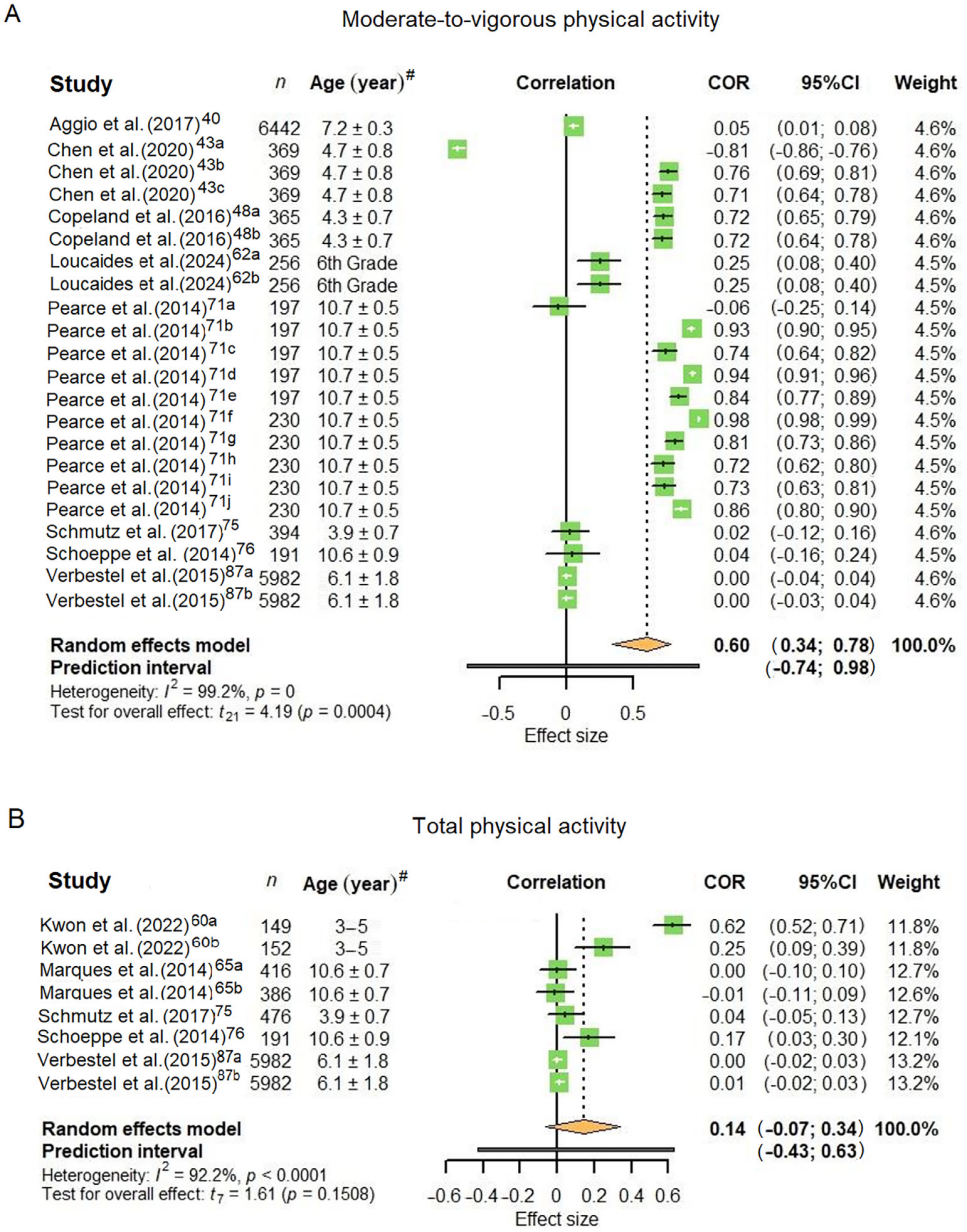
Forest plot of pooled effect sizes for (A) moderate-to-vigorous physical activity and (B) total physical activity. Entries labeled with suffixes (e.g., Chen et al. (2020)^43^^a^, Chen et al. (2020)^43^^b^, Chen et al. (2020)^43^^c^) represent distinct effect sizes reported within the same study. Detailed descriptions of these effect sizes are provided in Supplementary Table 3. ^#^Age are presented as mean ± SD except for Loucaides et al. (2024)^62^ which only provided information on grade level, and for Kwon et al. (2020)^60^ which provided the age range. 95%CI = 95% confidence interval; COR = correlation.

A meta-analysis was conducted for MVPA and total PA outcomes (Fig. 2). Details of the included studies and corresponding effect sizes can be found in Supplementary Table 6. For MVPA, 22 effect sizes from 8 studies^40^^,^^43^^,^^48^^,^^58^^,^^62^^,^^75^^,^^76^^,^^87^ were included in the analysis and showed an overall significant moderate positive correlation between AOP and MVPA (*r* = 0.60, 95% confidence interval (95%CI): 0.34–0.78, *p* = 0.0004; *I*^2^ = 99.2%). Eight effect sizes from 5 studies were included in the meta-analyses for total PA,^60^^,^^65^^,^^75^^,^^76^^,^^87^ which showed a weak positive correlation to AOP that was not statistically significant (*r* = 0.14, 95%CI: –0.07 to 0.34, *p* = 0.1508; *I*^2^ = 92.2%).

##### 3.3.1.2 Narrative synthesis

Among cross-sectional studies (*n* = 38),^39^^,^^40^^,^^42^^,^^43^^,^^47^^,^^48^^,^^50^^,^^52^, ^53^, ^54^^,^^56^, ^57^, ^58^^,^^60^, ^61^, ^62^, ^63^, ^64^, ^65^, ^66^, ^67^, ^68^^,^^71^, ^72^, ^73^, ^74^, ^75^, ^76^, ^77^^,^^79^^,^^80^^,^^82^, ^83^, ^84^^,^^86^^,^^87^^,^^90^^,^^91^ there was consistent evidence demonstrating increased AOP was related to increased light PA (5/6; 83.3%), MVPA (18/24; 75%), steps per day (3/3; 100%), and any intensity PA (11/15; 73.3%). There was inconsistent evidence to support a favorable relationship between AOP and meeting PA guidelines (3/6; 50%). No unfavorable associations were reported.

**Fig. 3:**
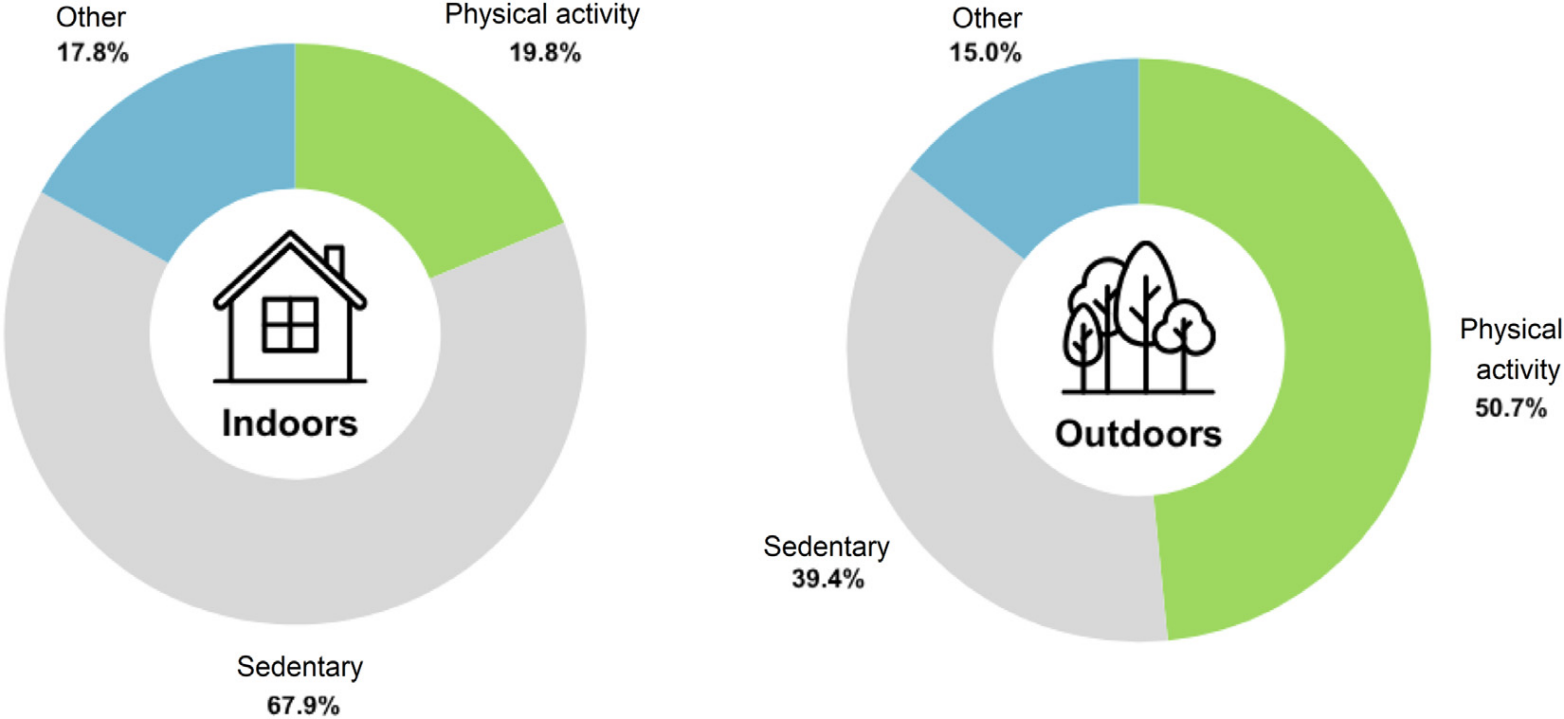
Percentage of time spent in sedentary behavior and physical activity during indoor and outdoor periods. Percentages do not add up to 100% as not all studies reported both physical activity and sedentary time. Values are rounded to the nearest tenth.

Eight cross-sectional studies directly compared outdoor PA levels and sedentary behavior to indoor PA levels using a combination of accelerometry and observation or Global Positioning System (GPS).^39^^,^^42^^,^^47^^,^^80^^,^^82^^,^^83^^,^^86^^,^^90^ On average, children spent 50.7% of their time outdoors engaging in PA compared to only 19.8% indoors (Fig. 3).

Six longitudinal studies investigated the relationship between AOP and PA over time.^55^^,^^59^^,^^67^^,^^70^^,^^78^^,^^89^ Study time periods ranged from 8 days to 32 years and showed consistent evidence that engaging in AOP had favorable longitudinal associations with total PA (3/4; 75%).^55^^,^^59^^,^^70^^,^^78^ There was inconsistent evidence for favorable long-term associations between AOP and MVPA (1/2; 50%).^67^^,^^89^

Eight studies investigated the effect of an unstructured outdoor play intervention aimed at increasing opportunities for outdoor play on PA levels.^41^^,^^44^^,^^45^^,^^49^^,^^51^^,^^81^^,^^85^^,^^88^ These interventions included 3 “open streets” programs,^49^^,^^51^^,^^85^ 3 interventions aimed at increasing allotted time spent outside during childcare,^41^^,^^81^^,^^88^ and 2 interventions investigating the efficacy of nature/outdoor play prescriptions.^44^^,^^45^ Six interventions (75%), 3 of which were the “open streets” interventions, demonstrated a favorable effect of the intervention on at least one PA outcome. No study showed unfavorable associations.

#### 3.3.2 Sedentary behavior and screen time

##### 3.3.2.1 Meta-analyses

**Fig. 4:**
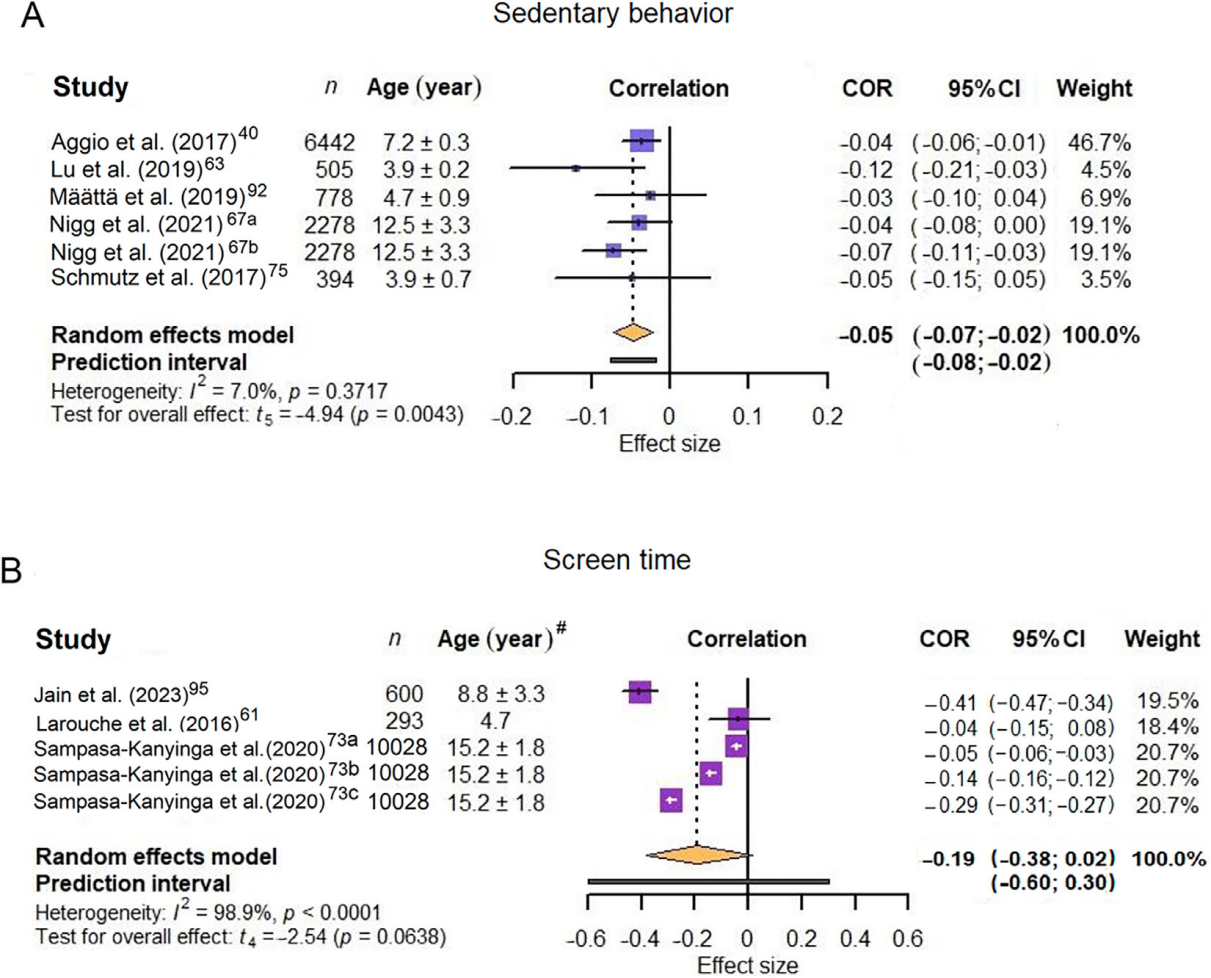
Forest plot of pooled effect sizes for (A) sedentary behavior and (B) screen time. Entries labeled with suffixes (e.g., Nigg et al. (2021)^67^^a^, Nigg et al. (2021)^67^^b^) represent distinct effect sizes reported within the same study. Detailed descriptions of these effect sizes are provided in Supplementary Table 3. ^#^Ages are presented as mean ± SD except for Larouche et al. (2016)^61^ which only provided a mean age. 95%CI = 95% confidence interval; COR = correlation.

Six associations from 5 individual studies^40^^,^^63^^,^^67^^,^^75^^,^^92^ were included in the meta-analyses for sedentary behavior, and 5 effect sizes from three studies^61^^,^^73^^,^^93^ for screen time (Fig. 4). Details of the included studies and corresponding effect sizes can be found in Supplementary Table 6. For sedentary behavior, a weak but significant negative correlation was identified (*r* = –0.05, 95%CI: –0.07 to −0.02, *p* < 0.0043; *I*^2^ = 7.0%). The correlation findings for screen time were non-significant (*r* = –0.19, 95%CI: –0.38 to 0.02, *p* = 0.0638; *I*^2^ = 98.9%).

##### 3.3.2.2 Narrative synthesis

For sedentary behavior, there was consistent evidence (12/18; 66.7%) across the cross-sectional study designs that engaging in more AOP was related to decreased sedentary behavior. No studies showed unfavorable associations. Two cross-sectional studies examined the relationship between AOP and screen time and showed a favorable association. For screen time guidelines, there was consistent evidence among the 3 cross-sectional studies that those who engaged in more AOP were more likely to meet screen time guidelines (2/3; 66.7%). Two studies^67^^,^^94^ examined the longitudinal associations between AOP and screen time; both showed mixed effects, including a mix of favorable and null findings in one study and favorable and unfavorable findings in the other. Six studies compared the amount of sedentary behavior when outdoors compared to indoors.^39^^,^^42^^,^^80^^,^^82^^,^^83^^,^^86^ Based on the average percentages reported, children engaged in sedentary behavior, on average, 39.4% of the time when outdoors compared to 67.9% of the time when indoors (Fig. 3).

Four studies^44^^,^^51^^,^^81^^,^^85^ examined the impact of an unstructured outdoor play intervention on sedentary behavior. Two interventions consisted of an “open streets” program^51^^,^^85^ and both showed at least 1 favorable effect on reducing sedentary behavior. One intervention focused on increasing outdoor play opportunities in childcare^81^ and another on physician-prescribed outdoor play,^44^ but neither showed an effect on sedentary behavior.

#### 3.3.3 Sleep

Eight studies^53^^,^^64^^,^^69^^,^^73^^,^^95^, ^96^, ^97^, ^98^ investigated the relationship between AOP and sleep, all of which employed a cross-sectional design (Table 2).

##### 3.3.3.1 Meta-analyses

**Fig. 5:**
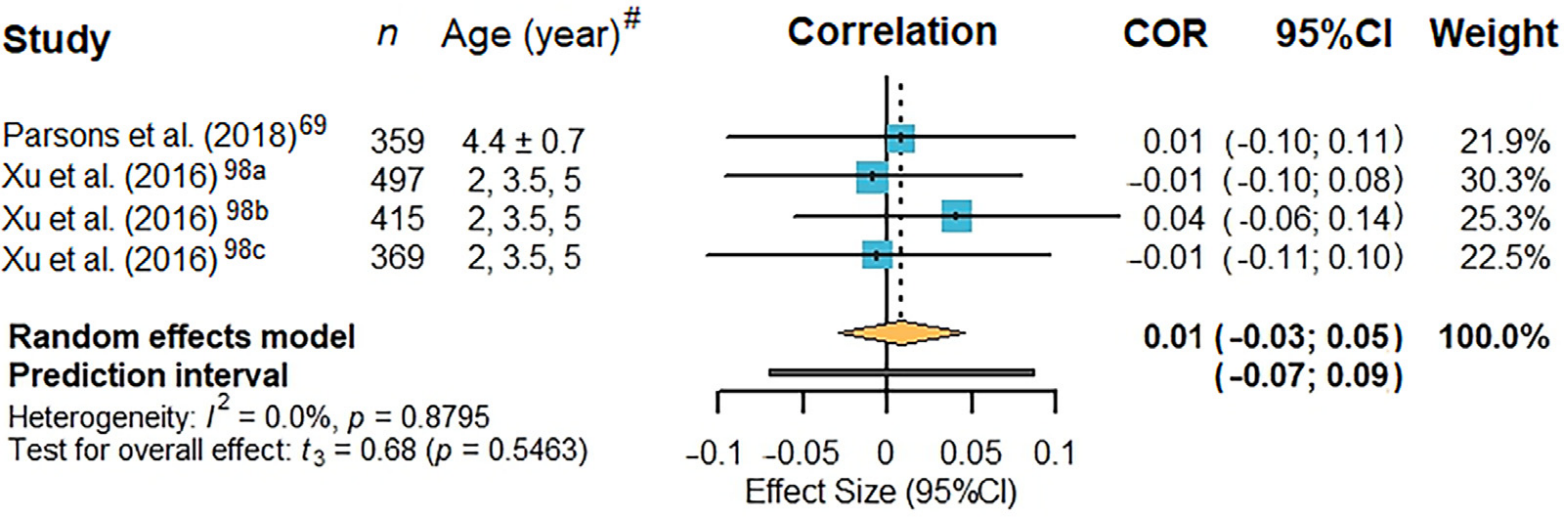
Forest plot of pooled effect sizes for sleep quantity. Entries labeled with suffixes (e.g., Xu et al. (2016)^98^^a^, Xu et al. (2016)^98^^b^, and Xu et al. (2016)^98^^c^) represent distinct effect sizes reported within the same study. Detailed descriptions of these effect sizes are provided in Supplementary Table 3. ^#^Ages are presented as mean ± SD except for Xu et al. (2016)^98^ which provided exact age groups. 95%CI = 95% confidence interval; COR = correlation.

Four associations from 2 individual studies^69^^,^^98^ were included in the meta-analyses for sleep (Fig. 5). Details of the included studies and corresponding effect sizes can be found in Supplementary Table 6. A non-significant, negligible correlation was identified for sleep and AOP (*r* = –0.01, 95%CI: –0.03 to 0.05, *p* = 0.8795; *I*^2^ = 0.0%).

##### 3.3.3.2 Narrative synthesis

Two studies examined sleep quality, which included “how rested one felt in the morning” and the “mood they woke up in”,^95^ earlier bedtime, and waking during the night.^98^ Both studies showed that engaging in more AOP was associated with better sleep quality. Three studies^69^^,^^96^^,^^98^ examined sleep quantity as an outcome. There was inconsistent evidence demonstrating a favorable association between AOP and sleep quantity (i.e., 1/3, 33% favorable). One study showed a favorable association between AOP and sleep duration,^96^ 1 study showed mixed associations (favorable and null),^98^ and 1 study showed no association.^69^ Four studies examined the relationship between AOP and meeting sleep guidelines. One study showed a favorable association,^97^ one study showed mixed associations (favorable and null),^73^ and two studies showed no association.^53^^,^^64^ No studies showed unfavorable associations.

## 4 Discussion

### 4.1 Relationship between AOP and movement behaviors

Our systematic review and meta-analyses examined the direct relationships between AOP and 24-h movement behaviors and identified substantially more papers focusing on sedentary behavior (*n* = 21) compared to Gray et al.^18^ (*n* = 4). We also included papers specifically investigating screen time, a key component of sedentary behavior,^99^ as well as 8 papers addressing sleep, a movement behavior absent from previous reviews (Gray et al.^18^ and others^100^^,^^101^).

Research devoted to examining the association between AOP and children’s movement behaviors has substantially expanded over the past decade. Our review confirms that a generally favorable relationship between AOP and PA exists, particularly for MVPA. That is, engaging in more AOP is related to an increase in the PA movement behavior dimension. Our meta-analyses revealed a moderate, statistically significant association for this relationship, reinforcing the robust and consistent link between AOP and MVPA. Importantly, it also extends the literature by including studies on light-intensity PA, an important but often-overlooked component of the activity spectrum.^3^^,^^102^^,^^103^

Six studies specifically examined the relationship between AOP and adherence to PA guidelines, with mixed findings.^61^^,^^64^^,^^72^, ^73^, ^74^^,^^77^ These mixed findings may reflect variability in how AOP and guideline adherence were operationalized across studies, or they could reflect the influence of contextual factors like the duration and quality of outdoor play, environmental supports, and social dynamics, suggesting that while AOP can be an important contributor to overall activity levels, it is not the sole determinant of whether children meet daily PA recommendations. This relationship was further demonstrated in seven studies reporting that more PA occurred when children were outside, although some PA was also occurring indoors.^39^^,^^42^^,^^47^^,^^80^^,^^82^^,^^83^^,^^86^ In the studies that met our inclusion criteria, we found clear evidence that providing children with more opportunities to play outside increases their engagement in PA above amounts normally observed indoors.

This review included both cross-sectional and longitudinal studies, providing insight into acute and temporal relationships between AOP and PA. Cross-sectional studies largely supported a favorable relationship, with nearly three-quarters reporting significant positive associations. Evidence from the small number of longitudinal studies suggested that AOP is associated with increased PA over time. This finding aligns with broader literature showing that outdoor environments support higher levels of PA duration among children and youth.^61^^,^^104^

In terms of sedentary behavior, the evidence consistently demonstrated that engaging in more AOP was related to less time spent in sedentary behavior. Across the included studies, no evidence suggested that AOP was associated with increased sedentary time, and most findings concluded AOP was a protective factor against sedentary behavior in children and youth. Although evidence on screen time was more limited in terms of number of studies and mixed findings, several studies reiterated that children who engage in more AOP are more likely to meet recommended screen time guidelines. Longitudinal evidence on screen use was particularly sparse and somewhat inconsistent, potentially due to pandemic-related shifts in children’s screen habits.^105^^,^^106^ The relationship evidenced between AOP and sleep included fewer studies but still offers early insight into potential benefits of the outdoor movement behavior. Two studies reported favorable associations between AOP and sleep quality,^95^^,^^98^ whereas findings for sleep duration were mixed. Some evidence suggested that children who engage in more outdoor play are more likely to meet sleep guidelines and sleep longer.^96^^,^^98^ These findings align with broader research linking PA and time spent outdoors to improved sleep outcomes in children,^107^^,^^108^ suggesting that outdoor exposure can support healthy sleep patterns in several ways, like increased PA and greater daylight exposure.

### 4.2 Intervention study key takeaways

Although limited in number, the 7 intervention studies included in this review support AOP as a low-cost, scalable strategy to improve movement behaviors in children. Public space interventions—such as Play Streets in Belgium,^51^ Ciclovía in Brazil,^85^ and Juega en tu Barrio in Chile^49^—showed the most promise, with reported increases in MVPA and reductions in sedentary time during intervention periods. In contrast, interventions in childcare settings, such as adding outdoor recess or offering unrestricted outdoor access, did not result in significant changes to PA or sedentary behavior in the studies identified in this review. Similarly, early evidence from nature prescription programs were mixed, suggesting that encouraging AOP alone may not be sufficient to meaningfully shift movement patterns. It is important to note that these intervention studies primarily examined changes based on assignment to intervention group, rather than actual changes in AOP behavior, limiting the conclusions that can be drawn in terms of the purposes of this review. Overall, their findings suggest that while AOP is indeed an appealing and accessible behavioral target strategy, its effectiveness likely depends on how interventions are designed in terms of their ability to shape the physical and social environments in which they are implemented and how they aim to actively promote physical movement behavior changes. To improve 24-h movement behaviors across diverse contexts, more research is needed to evaluate which intervention strategies most effectively increase AOP.

Despite growing interest in understanding how time spent in various movement behaviors interacts across the 24-h day,^109^ most studies in our review examined the three individual behaviors in isolation. This approach limits our ability to assess the holistic impact of AOP on the integrated nature of children’s daily activity. Compositional analysis—an approach that considers the finite nature of time and the interdependence of movement behaviors—is essential to fully capture how changes in one behavior (e.g., increasing AOP or PA) invariably displaces time from the other two domains (i.e., sedentary behavior and/or sleep). Future research employing compositional methods is needed to fully examine the mechanisms behind any redistribution of time across movement behaviors and to better elucidate any trade-offs or synergies AOP exerts within a 24-h framework.

### 4.3 Study limitations and future directions

This systematic review and meta-analyses provide unique insight into the relationship between AOP and 24-h movement behaviors, although several limitations should be considered when interpreting these findings. First, although many studies met our eligibility criteria, only a small subset were included in the meta-analyses due to high study heterogeneity driven, largely, by inconsistent definitions and varied measurement approaches to outdoor play. Defining whether activities were truly “play” (i.e., intrinsically motivated, voluntary, fun) is therefore not possible within the scope of this work. For screen time and sleep, there were fewer than 5 effect sizes included. Secondly, the GRADE assessment reflected an urgent need for more standardized, valid, and reliable measures to assess outdoor play and to improve comparability and evidence quality within and between investigations. The overall certainty of evidence was rated as low to very low, highlighting a need for more rigorous, well-designed studies with transparent and standardized reporting to support more confident and actionable recommendations. Third, although this review aimed to include people of all age groups, no studies that focused on adults met our inclusion criteria. This may be a result of stricter definitions used to distinguish “outdoor play” from “general PA”, making it difficult to identify outdoor play in the adult literature. Despite “play” being central to the conceptual foundation of the AOP Position Statement,^27^^,^^28^ it is possible that adult populations were not captured due to the limited use of the term “play” in adult population literature. To advance AOP research in adult populations, future work could explicitly examine adult experiences that align with the principles of play (e.g., intrinsically motivated), even when not labeled as “play”, to gain a better conceptual understanding of what “play” means for adults. Further, promoting the language of “play” in adult-focused research, practice, and policy may help to reframe the concept of play as being only for children, thus helping to better capture AOP happening among adults. Future research examining the relationship between AOP and movement behaviors among adults and older adults is needed to understand the potential for AOP to promote healthy movement behaviors for the whole population. In addition, due to limited heterogeneity among studies included in the meta-analysis, it was not possible to examine effects of AOP by age group (e.g., early years, school-aged, adolescents). Future research should explore potential age-related differences as more evidence becomes available. Finally, none of the included studies disaggregated data by equity-deserving groups. As outdoor environments constitute potentially inclusive, low-cost spaces for bodily movement, future research must prioritize equity-informed approaches and disaggregated analyses to assess whether AOP opportunities are equitably experienced and accessible for all.

## 5 Conclusion

This systematic review and meta-analysis updates evidence on the relationship between AOP and 24-h movement behaviors in children and youth. Positive associations were confirmed between AOP and PA, specifically for MVPA, and favorable outcomes were established for sedentary behavior, screen time, and sleep. The inclusion of meta-analytic data and a broader evidence base—including longitudinal and intervention studies—adds depth to the strength of and confidence of our analysis. Although some limitations in measurement techniques and study quality do persist within the cited studies, the overall body of evidence supports AOP as a low-cost, scalable strategy to promote healthy movement behaviors in children and youth. Future research should prioritize high-quality, longitudinal and experimental designs with standardized measures and use compositional data analyses to explore the importance of AOP across the lifespan and in diverse contexts such as public health, epidemiology, and education.

## Authors’ contributions

MEJ contributed to conceptualization, methodology, literature search, screening and selection, data extraction, risk of bias assessment, data analysis, GRADE assessment, writing – original draft, and project administration; LdL contributed to conceptualization, methodology, screening and selection, writing – original draft, and funding acquisition; EYL, SAM, and AP contributed to conceptualization, methodology, and data analysis/meta-analysis; LS contributed to conceptualization, methodology, and literature search; MST contributed to conceptualization, methodology, literature search, supervision, project administration, and funding acquisition; LMB contributed to conceptualization, methodology, and risk of bias assessment; OL and PBa contributed to methodology, screening and selection, and data extraction; AJ and JBS contributed to screening and selection and risk of bias assessment; EW and RF contributed to GRADE assessment; SR contributed to data extraction; TM, LMV, PBe, and SD contributed to screening and selection and data extraction; LM, PT, LJW, and VC contributed to screening and selection. All authors contributed to writing, reviewing, and editing of this manuscript. All authors have read and approved the final version of the manuscript, and agree with the order of presentation of the authors.

## Declaration of competing interest

MST is the President of Outdoor Play Canada and serves in this capacity as a volunteer. All authors declare that they have no competing interests.

## Supplementary materials

Image, application 1

Image, application 2

Image, application 3

Image, application 4

Image, application 5

Image, application 6

Image, video 7 dummy alt text

## Footnotes

Supplementary materials associated with this article can be found in the online version at doi:10.1016/j.jshs.2025.101115.
