# Supplementary material for: A systematic review and meta-analyses of the relationships between active outdoor play and 24-hour movement behaviors
Source: J Sport Health Sci. 2025 Dec 29;15:101115. doi: 10.1016/j.jshs.2025.101115 (PMC13053787; doi:10.1016/j.jshs.2025.101115)
Supplement: Supplementary file 2 [file mmc2.docx]

**Appendix B – Search Strategies**

**Medline**
1. (exp Environment/ or Parks, Recreational/ or (outside or outdoor* or play ground* or playground* or yard* or (school adj ground*) or green space* or greenspace* or blue space* or bluespace* or wilderness or natur* or forest* or park or parks or back yard* or backyard* or environment* or land-based or water-based or beach or bush).ti,ab.) adj1 (exp Exercise/ or exp "Play and Playthings"/ or ((physical adj activity) or active or exercis* or sport* or leisure or recreation* or adventur* or movement or play* or game or games or gym or gyms).ti,ab.)

2. Exercise/ or exp Accelerometry/

3. ((physical adj activit*) or exercis* or (physical adj inactivit*) or fitness or accelerometr* or actigraphy or "step count*" or pedomet*).ti,ab.

4. Sedentary Behavior/

5. (sedentary adj1 (behavi* or lifestyle* or life style*)).ti,ab.

6. exp Cell Phone/ or Screen Time/ or Video Games/ or Television/ or Computers/ or Microcomputers/

7. (screen or television or video gam* or laptop* or computer* or phone* or cellphone* or mobile device* or smartphone* or text messag* or texting).ti,ab.

8. exp Sleep/

9. (sleep* or nap or naps or napping or bedtime* or waketime*).ti,ab.

10. 24-hour movement behavio#r*.ti,ab.

11. or/2-10

12. (Randomized Controlled Trial or Controlled Clinical Trial or Pragmatic Clinical Trial or Equivalence Trial or Clinical Trial, Phase III).pt.

13. Randomized Controlled Trial/

14. exp Randomized Controlled Trials as Topic/

15. Controlled Clinical Trial/

16. exp Controlled Clinical Trials as Topic/

17. Randomization/

18. Random Allocation/

19. Double-Blind Method/ or Double Blind Procedure/ or Double-Blind Studies/ or Single-Blind Method/ or Single Blind Procedure/ or Single-Blind Studies/ or Placebos/ or Placebo/ or Control Groups/ or Control Group/

20. (random* or sham or placebo*).ti,ab,hw,kf.

21. ((singl* or doubl*) adj (blind* or dumm* or mask*)).ti,ab,hw,kf.

22. ((tripl* or trebl*) adj (blind* or dumm* or mask*)).ti,ab,hw,kf.

23. (control* adj3 (study or studies or trial* or group*)).ti,ab,kf.

24. (Nonrandom* or non random* or non-random* or quasi-random* or quasirandom*).ti,ab,hw,kf.

25. allocated.ti,ab,hw.

26. ((open label or open-label) adj5 (study or studies or trial*)).ti,ab,hw,kf.

27. ((equivalence or superiority or non-inferiority or noninferiority) adj3 (study or studies or trial*)).ti,ab,hw,kf.

28. (pragmatic study or pragmatic studies).ti,ab,hw,kf.

29. ((pragmatic or practical) adj3 trial*).ti,ab,hw,kf.

30. ((quasiexperimental or quasi-experimental) adj3 (study or studies or trial*)).ti,ab,hw,kf.

31. (phase adj3 (III or "3") adj3 (study or studies or trial*)).ti,hw,kf.

32. or/12-31

33. Epidemiologic Methods/

34. exp Epidemiologic Studies/

35. Observational Studies as Topic/

36. Clinical Studies as Topic/

37. case reports as topic/

38. (Observational Study or Validation Studies or Clinical Study).pt.

39. (observational adj3 (study or studies or design or analysis or analyses)).ti,ab,kf.

40. cohort*.ti,ab,kf.

41. (prospective adj7 (study or studies or design or analysis or analyses)).ti,ab,kf.

42. ((follow up or followup) adj7 (study or studies or design or analysis or analyses)).ti,ab,kf.

43. ((longitudinal or longterm or (long adj term)) adj7 (study or studies or design or analysis or analyses or data)).ti,ab,kf.

44. (retrospective adj7 (study or studies or design or analysis or analyses or data or review)).ti,ab,kf.

45. ((case adj control) or (case adj comparison) or (case adj controlled)).ti,ab,kf.

46. (case-referent adj3 (study or studies or design or analysis or analyses)).ti,ab,kf.

47. (population adj3 (study or studies or analysis or analyses)).ti,ab,kf.

48. (descriptive adj3 (study or studies or design or analysis or analyses)).ti,ab,kf.

49. ((multidimensional or (multi adj dimensional)) adj3 (study or studies or design or analysis or analyses)).ti,ab,kf.

50. (cross adj sectional adj7 (study or studies or design or research or analysis or analyses or survey or findings)).ti,ab,kf.

51. ((natural adj experiment) or (natural adj experiments)).ti,ab,kf.

52. (quasi adj (experiment or experiments or experimental)).ti,ab,kf.

53. ((non experiment or nonexperiment or non experimental or nonexperimental) adj3 (study or studies or design or analysis or analyses)).ti,ab,kf.

54. (prevalence adj3 (study or studies or analysis or analyses)).ti,ab,kf.

55. case series.ti,ab,kf.

56. case reports.pt.

57. (case adj3 (report or reports or study or studies or histories)).ti,ab,kf.

58. organizational case studies/

59. or/33-58

60. 32 or 59

61. 1 and 11 and 60

**Embase**

1. (recreational park/ or (outside or outdoor* or play ground* or playground* or yard* or (school adj ground*) or green space* or greenspace* or blue space* or bluespace* or wilderness or natur* or forest* or park or parks or back yard* or backyard* or environment* or land-based or water-based or beach or bush).ti,ab.) adj1 (Exercise/ or recreation/ or physical activity/ or ((physical adj activity) or active or exercis* or sport* or leisure or recreation* or adventur* or movement or play* or game or games or gym or gyms).ti,ab.)

2. accelerometer/ or accelerometry/

3. exercise/

4. ((physical adj activit*) or exercis* or (physical adj inactivit*) or fitness or accelerometr* or actigraphy or "step count*" or pedomet*).ti,ab.

5. sedentary lifestyle/

6. (sedentary adj1 (behavi* or lifestyle* or life style*)).ti,ab.

7. exp mobile phone/

8. screen time/

9. video game/

10. television/

11. computer/ or microcomputer/

12. text messaging/

13. (screen or television or video gam* or laptop* or computer* or phone* or cellphone* or mobile device* or smartphone* or text messag* or texting).ti,ab.

14. sleep/

15. (sleep* or nap or naps or napping or bedtime* or waketime*).ti,ab.

16. 24-hour movement behavio#r*.ti,ab.

17. or/2-16

18. Randomized Controlled Trial/

19. exp Randomized Controlled Trials as Topic/

20. Controlled Clinical Trial/

21. exp Controlled Clinical Trials as Topic/

22. Randomization/

23. Random Allocation/

24. Double-Blind Method/ or Double Blind Procedure/ or Double-Blind Studies/ or Single-Blind Method/ or Single Blind Procedure/ or Single-Blind Studies/ or Placebos/ or Placebo/ or Control Groups/ or Control Group/

25. (random* or sham or placebo*).ti,ab,hw,kf.

26. ((singl* or doubl*) adj (blind* or dumm* or mask*)).ti,ab,hw,kf.

27. ((tripl* or trebl*) adj (blind* or dumm* or mask*)).ti,ab,hw,kf.

28. (control* adj3 (study or studies or trial* or group*)).ti,ab,kf.

29. (Nonrandom* or non random* or non-random* or quasi-random* or quasirandom*).ti,ab,hw,kf.

30. allocated.ti,ab,hw.

31. ((open label or open-label) adj5 (study or studies or trial*)).ti,ab,hw,kf.

32. ((equivalence or superiority or non-inferiority or noninferiority) adj3 (study or studies or trial*)).ti,ab,hw,kf.

33. (pragmatic study or pragmatic studies).ti,ab,hw,kf.

34. ((pragmatic or practical) adj3 trial*).ti,ab,hw,kf.

35. ((quasiexperimental or quasi-experimental) adj3 (study or studies or trial*)).ti,ab,hw,kf.

36. (phase adj3 (III or "3") adj3 (study or studies or trial*)).ti,hw,kf.

37. or/18-36

38. observational study/

39. cohort analysis/

40. longitudinal study/

41. follow up/

42. retrospective study/

43. exp case control study/

44. cross-sectional study/

45. quasi experimental study/

46. prospective study/

47. (observational adj3 (study or studies or design or analysis or analyses)).ti,ab,kf.

48. cohort*.ti,ab,kf.

49. (prospective adj7 (study or studies or design or analysis or analyses)).ti,ab,kf.

50. ((follow up or followup) adj7 (study or studies or design or analysis or analyses)).ti,ab,kf.

51. ((longitudinal or longterm or (long adj term)) adj7 (study or studies or design or analysis or analyses or data)).ti,ab,kf.

52. (retrospective adj7 (study or studies or design or analysis or analyses or data or review)).ti,ab,kf.

53. ((case adj control) or (case adj comparison) or (case adj controlled)).ti,ab,kf.

54. (case-referent adj3 (study or studies or design or analysis or analyses)).ti,ab,kf.

55. (population adj3 (study or studies or analysis or analyses)).ti,ab,kf.

56. (descriptive adj3 (study or studies or design or analysis or analyses)).ti,ab,kf.

57. ((multidimensional or (multi adj dimensional)) adj3 (study or studies or design or analysis or analyses)).ti,ab,kf.

58. (cross adj sectional adj7 (study or studies or design or research or analysis or analyses or survey or findings)).ti,ab,kf.

59. ((natural adj experiment) or (natural adj experiments)).ti,ab,kf.

60. (quasi adj (experiment or experiments or experimental)).ti,ab,kf.

61. ((non experiment or nonexperiment or non experimental or nonexperimental) adj3 (study or studies or design or analysis or analyses)).ti,ab,kf.

62. (prevalence adj3 (study or studies or analysis or analyses)).ti,ab,kf.

63. case series.ti,ab,kf.

64. case study/

65. case report/

66. (case adj3 (report or reports or study or studies or histories)).ti,ab,kf.

67. or/38-66

68. 37 or 67

69. 1 and 17 and 68

**CINAHL**

**Search History**

| \| **#** \| **Query** \| \| --- \| --- \| \| S26 \| S3 AND S11 AND S25 \| \| S25 \| S12 OR S13 OR S14 OR S15 OR S16 OR S17 OR S18 OR S19 OR S20 OR S21 OR S22 OR S23 OR S24 \| \| S24 \| TI 24-hour movement behav* OR AB 24-hour movement behav* \| \| S23 \| TI ( (sleep* or nap or naps or napping or bedtime* or waketime*) ) OR AB ( (sleep* or nap or naps or napping or bedtime* or waketime*) ) \| \| S22 \| (MH "Sleep") \| \| S21 \| TI ( (screen or television or video gam* or laptop* or computer* or phone* or cellphone* or mobile device* or smartphone* or text messag* or texting) ) OR AB ( (screen or television or video gam* or laptop* or computer* or phone* or cellphone* or mobile device* or smartphone* or text messag* or texting) ) \| \| S20 \| (MH "Television") OR (MH "Text Messaging") \| \| S19 \| (MH "Video Games") \| \| S18 \| (MH "Cellular Phone") OR (MH "Text Messaging") OR (MH "Smartphone") \| \| S17 \| TI ( (sedentary N2 (behavi* or lifestyle* or life style*)) ) OR AB ( (sedentary N2 (behavi* or lifestyle* or life style*)) ) \| \| S16 \| (MH "Life Style, Sedentary") OR (MH "Screen Time") \| \| S15 \| TI ( ((physical adj activit*) or exercis* or (physical adj inactivit*) or fitness or accelerometr* or actigraphy or "step count*" or pedomet*) ) OR AB ( ((physical adj activit*) or exercis* or (physical adj inactivit*) or fitness or accelerometr* or actigraphy or "step count*" or pedomet*) ) \| \| S14 \| (MH "Physical Activity") \| \| S13 \| (MH "Accelerometry+") \| \| S12 \| (MH "Exercise") \| \| S11 \| S9 N2 S10 \| \| S10 \| S6 OR S7 OR S8 \| \| S9 \| S4 OR S5 \| \| S8 \| TI ( ((physical adj activity) or active or exercis* or sport* or leisure or recreation* or adventur* or movement or play* or game or games or gym or gyms) ) OR AB ( ((physical adj activity) or active or exercis* or sport* or leisure or recreation* or adventur* or movement or play* or game or games or gym or gyms) ) \| \| S7 \| (MH "Recreation+") \| \| S6 \| (MH "Exercise+") OR (MH "Physical Activity") \| \| S5 \| TI ( (outside or outdoor* or play ground* or playground* or yard* or (school adj ground*) or green space* or greenspace* or blue space* or bluespace* or wilderness or natur* or forest* or park or parks or back yard* or backyard* or environment* or land-based or water-based or beach or bush) ) OR AB ( (outside or outdoor* or play ground* or playground* or yard* or (school adj ground*) or green space* or greenspace* or blue space* or bluespace* or wilderness or natur* or forest* or park or parks or back yard* or backyard* or environment* or land-based or water-based or beach or bush) ) \| \| S4 \| (MH "Environment") \| \| S3 \| S1 OR S2 \| \| S2 \| ((MH "Experimental Studies+") OR (MH "Multicenter Studies") OR (MH "Random Sample+") OR (MH "Placebos") OR (MH "Control (Research)+") OR (MH "Crossover Design") OR ((TI random* OR AB random*) OR (TI sham OR AB sham) OR (TI placebo* OR AB placebo*)) OR (((TI singl* OR AB singl*) OR (TI doubl* OR AB doubl*)) W1 ((TI blind* OR AB blind*) OR (TI dumm* OR AB dumm*) OR (TI mask* OR AB mask*))) OR (((TI tripl* OR AB tripl*) OR (TI trebl* OR AB trebl*)) W1 ((TI blind* OR AB blind*) OR (TI dumm* OR AB dumm*) OR (TI mask* OR AB mask*))) OR ((TI control* OR AB control*) N3 ((TI study OR AB study) OR (TI studies OR AB studies) OR (TI trial* OR AB trial*) OR (TI group* OR AB group*))) OR ((TI clinical OR AB clinical) N3 ((TI study OR AB study) OR (TI studies OR AB studies) OR (TI trial* OR AB trial*))) OR ((TI Nonrandom* OR AB Nonrandom*) OR (TI "non random*" OR AB "non random*") OR (TI "non-random*" OR AB "non-random*") OR (TI "quasi-random*" OR AB "quasi-random*") OR (TI quasirandom* OR AB quasirandom*)) OR ((TI phase OR AB phase) N6 ((TI study OR AB study) OR (TI studies OR AB studies) OR (TI trial* OR AB trial*))) OR (((TI crossover OR AB crossover) OR (TI "cross-over" OR AB "cross-over")) N3 ((TI study OR AB study) OR (TI studies OR AB studies) OR (TI trial* OR AB trial*))) OR (((TI multicent* OR AB multicent*) OR (TI "multi-cent*" OR AB "multi-cent*")) N3 ((TI study OR AB study) OR (TI studies OR AB studies) OR (TI trial* OR AB trial*))) OR (TI allocated OR AB allocated) OR (((TI "open label" OR AB "open label") OR (TI "open-label" OR AB "open-label")) N5 ((TI study OR AB study) OR (TI studies OR AB studies) OR (TI trial* OR AB trial*))) OR (((TI equivalence OR AB equivalence) OR (TI superiority OR AB superiority) OR (TI "non-inferiority" OR AB "non-inferiority") OR (TI noninferiority OR AB noninferiority)) N3 ((TI study OR AB study) OR (TI studies OR AB studies) OR (TI trial* OR AB trial*))) OR ((TI "pragmatic study" OR AB "pragmatic study") OR (TI "pragmatic studies" OR AB "pragmatic studies")) OR (((TI pragmatic OR AB pragmatic) OR (TI practical OR AB practical)) N3 (TI trial* OR AB trial*)) OR (((TI quasiexperimental OR AB quasiexperimental) OR (TI "quasi-experimental" OR AB "quasi-experimental")) N3 ((TI study OR AB study) OR (TI studies OR AB studies) OR (TI trial* OR AB trial*))) OR (TI trial)) \| \| S1 \| (MH "Nonexperimental Studies+") OR (MH "Correlational Studies") OR (MH "Control Group") OR (MH "Matched-Pair Analysis") OR ((TI observational OR AB observational) N3 ((TI study OR AB study) OR (TI studies OR AB studies) OR (TI design OR AB design) OR (TI analysis OR AB analysis) OR (TI analyses OR AB analyses))) OR (TI cohort* OR AB cohort*) OR ((TI prospective OR AB prospective) N7 ((TI study OR AB study) OR (TI studies OR AB studies) OR (TI design OR AB design) OR (TI analysis OR AB analysis) OR (TI analyses OR AB analyses))) OR (((TI "follow up" OR AB "follow up") OR (TI followup OR AB followup)) N7 ((TI study OR AB study) OR (TI studies OR AB studies) OR (TI design OR AB design) OR (TI analysis OR AB analysis) OR (TI analyses OR AB analyses))) OR (((TI longitudinal OR AB longitudinal) OR (TI longterm OR AB longterm) OR ((TI long OR AB long) W1 (TI term OR AB term))) N7 ((TI study OR AB study) OR (TI studies OR AB studies) OR (TI design OR AB design) OR (TI analysis OR AB analysis) OR (TI analyses OR AB analyses) OR (TI data OR AB data))) OR ((TI retrospective OR AB retrospective) N7 ((TI study OR AB study) OR (TI studies OR AB studies) OR (TI design OR AB design) OR (TI analysis OR AB analysis) OR (TI analyses OR AB analyses) OR (TI data OR AB data) OR (TI review OR AB review))) OR (((TI case OR AB case) W1 (TI control OR AB control)) OR ((TI case OR AB case) W1 (TI comparison OR AB comparison)) OR ((TI case OR AB case) W1 (TI controlled OR AB controlled))) OR ((TI case-referent OR AB case-referent) N3 ((TI study OR AB study) OR (TI studies OR AB studies) OR (TI design OR AB design) OR (TI analysis OR AB analysis) OR (TI analyses OR AB analyses))) OR ((TI population OR AB population) N3 ((TI study OR AB study) OR (TI studies OR AB studies) OR (TI analysis OR AB analysis) OR (TI analyses OR AB analyses))) OR ((TI descriptive OR AB descriptive) N3 ((TI study OR AB study) OR (TI studies OR AB studies) OR (TI design OR AB design) OR (TI analysis OR AB analysis) OR (TI analyses OR AB analyses))) OR (((TI multidimensional OR AB multidimensional) OR ((TI multi OR AB multi) W1 (TI dimensional OR AB dimensional))) N3 ((TI study OR AB study) OR (TI studies OR AB studies) OR (TI design OR AB design) OR (TI analysis OR AB analysis) OR (TI analyses OR AB analyses))) OR ((TI cross OR AB cross) W1 (TI sectional OR AB sectional) N7 ((TI study OR AB study) OR (TI studies OR AB studies) OR (TI design OR AB design) OR (TI research OR AB research) OR (TI analysis OR AB analysis) OR (TI analyses OR AB analyses) OR (TI survey OR AB survey) OR (TI findings OR AB findings))) OR (((TI natural OR AB natural) W1 (TI experiment OR AB experiment)) OR ((TI natural OR AB natural) W1 (TI experiments OR AB experiments))) OR ((TI quasi OR AB quasi) W1 ((TI experiment OR AB experiment) OR (TI experiments OR AB experiments) OR (TI experimental OR AB experimental))) OR (((TI "non experiment" OR AB "non experiment") OR (TI nonexperiment OR AB nonexperiment) OR (TI "non experimental" OR AB "non experimental") OR (TI nonexperimental OR AB nonexperimental)) N3 ((TI study OR AB study) OR (TI studies OR AB studies) OR (TI design OR AB design) OR (TI analysis OR AB analysis) OR (TI analyses OR AB analyses))) OR ((TI prevalence OR AB prevalence) N3 ((TI study OR AB study) OR (TI studies OR AB studies) OR (TI analysis OR AB analysis) OR (TI analyses OR AB analyses))) OR (TI "case series" OR AB "case series") OR ((TI case OR AB case) N3 ((TI report OR AB report) OR (TI reports OR AB reports) OR (TI study OR AB study) OR (TI studies OR AB studies) OR (TI histories OR AB histories))) OR MH ("case studies") \| |
| --- | --- | --- | --- | --- | --- | --- | --- | --- | --- | --- | --- | --- | --- | --- | --- | --- | --- | --- | --- | --- | --- | --- | --- | --- | --- | --- | --- | --- | --- | --- | --- | --- | --- | --- | --- | --- | --- | --- | --- | --- | --- | --- | --- | --- | --- | --- | --- | --- | --- | --- | --- | --- | --- | --- |
|  |

**SportDiscus**

**Search History**

| \| **#** \| **Query** \| \| --- \| --- \| \| S26 \| S3 AND S11 AND S25 \| \| S25 \| S12 OR S13 OR S14 OR S15 OR S16 OR S17 OR S18 OR S19 OR S20 OR S21 OR S22 OR S23 OR S24 \| \| S24 \| TI 24-hour movement behav* OR AB 24-hour movement behav* \| \| S23 \| TI ( (sleep* or nap or naps or napping or bedtime* or waketime*) ) OR AB ( (sleep* or nap or naps or napping or bedtime* or waketime*) ) \| \| S22 \| (MH "Sleep") \| \| S21 \| TI ( (screen or television or video gam* or laptop* or computer* or phone* or cellphone* or mobile device* or smartphone* or text messag* or texting) ) OR AB ( (screen or television or video gam* or laptop* or computer* or phone* or cellphone* or mobile device* or smartphone* or text messag* or texting) ) \| \| S20 \| (MH "Television") OR (MH "Text Messaging") \| \| S19 \| (MH "Video Games") \| \| S18 \| (MH "Cellular Phone") OR (MH "Text Messaging") OR (MH "Smartphone") \| \| S17 \| TI ( (sedentary N2 (behavi* or lifestyle* or life style*)) ) OR AB ( (sedentary N2 (behavi* or lifestyle* or life style*)) ) \| \| S16 \| (MH "Life Style, Sedentary") OR (MH "Screen Time") \| \| S15 \| TI ( ((physical adj activit*) or exercis* or (physical adj inactivit*) or fitness or accelerometr* or actigraphy or "step count*" or pedomet*) ) OR AB ( ((physical adj activit*) or exercis* or (physical adj inactivit*) or fitness or accelerometr* or actigraphy or "step count*" or pedomet*) ) \| \| S14 \| (MH "Physical Activity") \| \| S13 \| (MH "Accelerometry+") \| \| S12 \| (MH "Exercise") \| \| S11 \| S9 N2 S10 \| \| S10 \| S6 OR S7 OR S8 \| \| S9 \| S4 OR S5 \| \| S8 \| TI ( ((physical adj activity) or active or exercis* or sport* or leisure or recreation* or adventur* or movement or play* or game or games or gym or gyms) ) OR AB ( ((physical adj activity) or active or exercis* or sport* or leisure or recreation* or adventur* or movement or play* or game or games or gym or gyms) ) \| \| S7 \| (MH "Recreation+") \| \| S6 \| (MH "Exercise+") OR (MH "Physical Activity") \| \| S5 \| TI ( (outside or outdoor* or play ground* or playground* or yard* or (school adj ground*) or green space* or greenspace* or blue space* or bluespace* or wilderness or natur* or forest* or park or parks or back yard* or backyard* or environment* or land-based or water-based or beach or bush) ) OR AB ( (outside or outdoor* or play ground* or playground* or yard* or (school adj ground*) or green space* or greenspace* or blue space* or bluespace* or wilderness or natur* or forest* or park or parks or back yard* or backyard* or environment* or land-based or water-based or beach or bush) ) \| \| S4 \| (MH "Environment") \| \| S3 \| S1 OR S2 \| \| S2 \| ((MH "Experimental Studies+") OR (MH "Multicenter Studies") OR (MH "Random Sample+") OR (MH "Placebos") OR (MH "Control (Research)+") OR (MH "Crossover Design") OR ((TI random* OR AB random*) OR (TI sham OR AB sham) OR (TI placebo* OR AB placebo*)) OR (((TI singl* OR AB singl*) OR (TI doubl* OR AB doubl*)) W1 ((TI blind* OR AB blind*) OR (TI dumm* OR AB dumm*) OR (TI mask* OR AB mask*))) OR (((TI tripl* OR AB tripl*) OR (TI trebl* OR AB trebl*)) W1 ((TI blind* OR AB blind*) OR (TI dumm* OR AB dumm*) OR (TI mask* OR AB mask*))) OR ((TI control* OR AB control*) N3 ((TI study OR AB study) OR (TI studies OR AB studies) OR (TI trial* OR AB trial*) OR (TI group* OR AB group*))) OR ((TI clinical OR AB clinical) N3 ((TI study OR AB study) OR (TI studies OR AB studies) OR (TI trial* OR AB trial*))) OR ((TI Nonrandom* OR AB Nonrandom*) OR (TI "non random*" OR AB "non random*") OR (TI "non-random*" OR AB "non-random*") OR (TI "quasi-random*" OR AB "quasi-random*") OR (TI quasirandom* OR AB quasirandom*)) OR ((TI phase OR AB phase) N6 ((TI study OR AB study) OR (TI studies OR AB studies) OR (TI trial* OR AB trial*))) OR (((TI crossover OR AB crossover) OR (TI "cross-over" OR AB "cross-over")) N3 ((TI study OR AB study) OR (TI studies OR AB studies) OR (TI trial* OR AB trial*))) OR (((TI multicent* OR AB multicent*) OR (TI "multi-cent*" OR AB "multi-cent*")) N3 ((TI study OR AB study) OR (TI studies OR AB studies) OR (TI trial* OR AB trial*))) OR (TI allocated OR AB allocated) OR (((TI "open label" OR AB "open label") OR (TI "open-label" OR AB "open-label")) N5 ((TI study OR AB study) OR (TI studies OR AB studies) OR (TI trial* OR AB trial*))) OR (((TI equivalence OR AB equivalence) OR (TI superiority OR AB superiority) OR (TI "non-inferiority" OR AB "non-inferiority") OR (TI noninferiority OR AB noninferiority)) N3 ((TI study OR AB study) OR (TI studies OR AB studies) OR (TI trial* OR AB trial*))) OR ((TI "pragmatic study" OR AB "pragmatic study") OR (TI "pragmatic studies" OR AB "pragmatic studies")) OR (((TI pragmatic OR AB pragmatic) OR (TI practical OR AB practical)) N3 (TI trial* OR AB trial*)) OR (((TI quasiexperimental OR AB quasiexperimental) OR (TI "quasi-experimental" OR AB "quasi-experimental")) N3 ((TI study OR AB study) OR (TI studies OR AB studies) OR (TI trial* OR AB trial*))) OR (TI trial)) \| \| S1 \| (MH "Nonexperimental Studies+") OR (MH "Correlational Studies") OR (MH "Control Group") OR (MH "Matched-Pair Analysis") OR ((TI observational OR AB observational) N3 ((TI study OR AB study) OR (TI studies OR AB studies) OR (TI design OR AB design) OR (TI analysis OR AB analysis) OR (TI analyses OR AB analyses))) OR (TI cohort* OR AB cohort*) OR ((TI prospective OR AB prospective) N7 ((TI study OR AB study) OR (TI studies OR AB studies) OR (TI design OR AB design) OR (TI analysis OR AB analysis) OR (TI analyses OR AB analyses))) OR (((TI "follow up" OR AB "follow up") OR (TI followup OR AB followup)) N7 ((TI study OR AB study) OR (TI studies OR AB studies) OR (TI design OR AB design) OR (TI analysis OR AB analysis) OR (TI analyses OR AB analyses))) OR (((TI longitudinal OR AB longitudinal) OR (TI longterm OR AB longterm) OR ((TI long OR AB long) W1 (TI term OR AB term))) N7 ((TI study OR AB study) OR (TI studies OR AB studies) OR (TI design OR AB design) OR (TI analysis OR AB analysis) OR (TI analyses OR AB analyses) OR (TI data OR AB data))) OR ((TI retrospective OR AB retrospective) N7 ((TI study OR AB study) OR (TI studies OR AB studies) OR (TI design OR AB design) OR (TI analysis OR AB analysis) OR (TI analyses OR AB analyses) OR (TI data OR AB data) OR (TI review OR AB review))) OR (((TI case OR AB case) W1 (TI control OR AB control)) OR ((TI case OR AB case) W1 (TI comparison OR AB comparison)) OR ((TI case OR AB case) W1 (TI controlled OR AB controlled))) OR ((TI case-referent OR AB case-referent) N3 ((TI study OR AB study) OR (TI studies OR AB studies) OR (TI design OR AB design) OR (TI analysis OR AB analysis) OR (TI analyses OR AB analyses))) OR ((TI population OR AB population) N3 ((TI study OR AB study) OR (TI studies OR AB studies) OR (TI analysis OR AB analysis) OR (TI analyses OR AB analyses))) OR ((TI descriptive OR AB descriptive) N3 ((TI study OR AB study) OR (TI studies OR AB studies) OR (TI design OR AB design) OR (TI analysis OR AB analysis) OR (TI analyses OR AB analyses))) OR (((TI multidimensional OR AB multidimensional) OR ((TI multi OR AB multi) W1 (TI dimensional OR AB dimensional))) N3 ((TI study OR AB study) OR (TI studies OR AB studies) OR (TI design OR AB design) OR (TI analysis OR AB analysis) OR (TI analyses OR AB analyses))) OR ((TI cross OR AB cross) W1 (TI sectional OR AB sectional) N7 ((TI study OR AB study) OR (TI studies OR AB studies) OR (TI design OR AB design) OR (TI research OR AB research) OR (TI analysis OR AB analysis) OR (TI analyses OR AB analyses) OR (TI survey OR AB survey) OR (TI findings OR AB findings))) OR (((TI natural OR AB natural) W1 (TI experiment OR AB experiment)) OR ((TI natural OR AB natural) W1 (TI experiments OR AB experiments))) OR ((TI quasi OR AB quasi) W1 ((TI experiment OR AB experiment) OR (TI experiments OR AB experiments) OR (TI experimental OR AB experimental))) OR (((TI "non experiment" OR AB "non experiment") OR (TI nonexperiment OR AB nonexperiment) OR (TI "non experimental" OR AB "non experimental") OR (TI nonexperimental OR AB nonexperimental)) N3 ((TI study OR AB study) OR (TI studies OR AB studies) OR (TI design OR AB design) OR (TI analysis OR AB analysis) OR (TI analyses OR AB analyses))) OR ((TI prevalence OR AB prevalence) N3 ((TI study OR AB study) OR (TI studies OR AB studies) OR (TI analysis OR AB analysis) OR (TI analyses OR AB analyses))) OR (TI "case series" OR AB "case series") OR ((TI case OR AB case) N3 ((TI report OR AB report) OR (TI reports OR AB reports) OR (TI study OR AB study) OR (TI studies OR AB studies) OR (TI histories OR AB histories))) OR MH ("case studies") \| |
| --- | --- | --- | --- | --- | --- | --- | --- | --- | --- | --- | --- | --- | --- | --- | --- | --- | --- | --- | --- | --- | --- | --- | --- | --- | --- | --- | --- | --- | --- | --- | --- | --- | --- | --- | --- | --- | --- | --- | --- | --- | --- | --- | --- | --- | --- | --- | --- | --- | --- | --- | --- | --- | --- | --- |

**SCOPUS**

( ( ( TITLE-ABS-KEY ( observational W/3 ( study OR studies OR design OR analysis OR analyses ) ) OR TITLE-ABS-KEY ( cohort* ) OR TITLE-ABS-KEY ( prospective W/7 ( study OR studies OR design OR analysis OR analyses ) ) OR TITLE-ABS-KEY ( ( "follow up" OR followup ) W/7 ( study OR studies OR design OR analysis OR analyses ) ) OR TITLE-ABS-KEY ( ( longitudinal OR longterm OR ( long W/1 term ) ) W/7 ( study OR studies OR design OR analysis OR analyses OR data ) ) OR TITLE-ABS-KEY ( retrospective W/7 ( study OR studies OR design OR analysis OR analyses OR data OR review ) ) OR TITLE-ABS-KEY ( ( case W/1 control ) OR ( case W/1 comparison ) OR ( case W/1 controlled ) ) OR TITLE-ABS-KEY ( case-referent W/3 ( study OR studies OR design OR analysis OR analyses ) ) OR TITLE-ABS-KEY ( population W/3 ( study OR studies OR analysis OR analyses ) ) OR TITLE-ABS-KEY ( descriptive W/3 ( study OR studies OR design OR analysis OR analyses ) ) OR TITLE-ABS-KEY ( ( multidimensional OR ( multi W/1 dimensional ) ) W/3 ( study OR studies OR design OR analysis OR analyses ) ) OR TITLE-ABS-KEY ( cross W/1 sectional W/7 ( study OR studies OR design OR research OR analysis OR analyses OR survey OR findings ) ) OR TITLE-ABS-KEY ( ( natural W/1 experiment ) OR ( natural W/1 experiments ) ) OR TITLE-ABS-KEY ( quasi W/1 ( experiment OR experiments OR experimental ) ) OR TITLE-ABS-KEY ( ( "non experiment" OR nonexperiment OR "non experimental" OR nonexperimental ) W/3 ( study OR studies OR design OR analysis OR analyses ) ) OR TITLE-ABS-KEY ( prevalence W/3 ( study OR studies OR analysis OR analyses ) ) OR TITLE-ABS-KEY ( "case series" ) OR TITLE-ABS-KEY ( case W/3 ( report OR reports OR study OR studies OR histories ) ) ) ) OR ( ( TITLE-ABS-KEY ( random* OR sham OR placebo* ) OR TITLE-ABS-KEY ( ( singl* OR doubl* ) W/1 ( blind* OR dumm* OR mask* ) ) OR TITLE-ABS-KEY ( ( tripl* OR trebl* ) W/1 ( blind* OR dumm* OR mask* ) ) OR TITLE-ABS-KEY ( control* W/3 ( study OR studies OR trial* OR group* ) ) OR TITLE-ABS-KEY ( clinical W/3 ( study OR studies OR trial* ) ) OR TITLE-ABS-KEY ( nonrandom* OR "non random*" OR non-random* OR quasi-random* OR quasirandom* ) OR TITLE-ABS-KEY ( phase W/6 ( study OR studies OR trial* ) ) OR TITLE-ABS-KEY ( ( crossover OR cross-over ) W/3 ( study OR studies OR trial* ) ) OR TITLE-ABS-KEY ( ( multicent* OR multi-cent* ) W/3 ( study OR studies OR trial* ) ) OR TITLE-ABS ( allocated ) OR TITLE-ABS-KEY ( ( "open label" OR open-label ) W/5 ( study OR studies OR trial* ) ) OR TITLE-ABS-KEY ( ( equivalence OR superiority OR non-inferiority OR noninferiority ) W/3 ( study OR studies OR trial* ) ) OR TITLE-ABS-KEY ( "pragmatic study" OR "pragmatic studies" ) OR TITLE-ABS-KEY ( ( pragmatic OR practical ) W/3 trial* ) OR TITLE-ABS-KEY ( ( quasiexperimental OR quasi-experimental ) W/3 ( study OR studies OR trial* ) ) OR TITLE ( trial ) OR KEY ( trial ) ) ) ) AND ( ( ( TITLE-ABS-KEY ( 24-hour AND movement AND behav* ) ) OR ( TITLE-ABS-KEY ( sleep* OR nap OR naps OR napping OR bedtime* OR waketime* ) ) OR ( TITLE-ABS-KEY ( screen OR television OR video AND gam* OR laptop* OR computer* OR phone* OR cellphone* OR mobile AND device* OR smartphone* OR text AND messag* OR texting ) ) OR ( TITLE-ABS-KEY ( sedentary W/2 ( behav* OR lifestyle* OR style* ) ) ) OR ( TITLE-ABS-KEY ( ( ( physical AND activit* ) OR exercis* OR ( physical AND inactivit* ) OR fitness OR accelerometr* OR actigraphy OR "step count*" OR pedomet* ) ) ) ) AND ( TITLE-ABS-KEY ( ( outside OR outdoor* OR playground* OR yard* OR greenspace* OR bluespace* OR wilderness OR natur* OR forest* OR park OR parks OR backyard* OR environment* OR land-based OR water-based OR beach OR bush ) W/2 ( active OR exercis* OR sport* OR leisure OR recreation* OR adventur* OR movement OR play* OR game OR games OR gym OR gyms ) ) ) )
