# Supplementary material for: A systematic review and meta-analyses of the relationships between active outdoor play and 24-hour movement behaviors
Source: J Sport Health Sci. 2025 Dec 29;15:101115. doi: 10.1016/j.jshs.2025.101115 (PMC13053787; doi:10.1016/j.jshs.2025.101115)
Supplement: Supplementary file 3 [file mmc3.docx]

| **Appendix C – Study Characteristics** | | | | | | | | | | | |
| --- | --- | --- | --- | --- | --- | --- | --- | --- | --- | --- | --- |
|  |  |  |  |  |  |  |  | **Movement Behaviours** | | | |
| **Author, year** | **Country** | **Sample Size** | **% female** | **Age in Years**  **(M, SD)** | **Measure of AOP** | **Measure of Movement Behaviour** | **Study Design** | **Physical Activity** | **Sedentary Behaviour** | **Screen Time** | **Sleep** |
| Aggio 2017 | United Kingdom | 6442 | 49.3%; 51.1% | 7.2, ±0.3 | Parent report | Physical activity (PA): Accelerometer  Sedentary behaviour: Parent report | Cross-sectional | ✓ | ✓ |  |  |
| Alhassan 2007 | US | 32 | 37.5% | 3.6, ±0.5 | Direct observation | PA: Accelerometer | Randomized controlled trial | ✓ |  |  |  |
| Cerin 2016 | US | 66 | 42% | 4.5,±0.8 | Accelerometer/GPS | PA and sedentary behaviour: Accelerometer | Cross-sectional | ✓ | ✓ |  |  |
| Chen 2020 | Sweden | 369 | 45% | 4.7, ±0.8 | Teacher log | PA: Accelerometer | Cross-sectional | ✓ |  |  |  |
| Christiana 2017 | US | 70 | 44.3% | Not reported. | Parent report | PA and sedentary behaviour: Parent report | Quasi-experimental | ✓ | ✓ |  |  |
| Christiana 2023 | US | 78 | 57.7 % | 7.7, ±1 | Parent frequency report | PA: Parent report | Quasi-experimental | ✓ |  |  |  |
| Cleland 2008 | Australia | 548 | 53% | 5 to 6, 10 to 12 | Parent report | PA: Accelerometer | Longitudinal | ✓ |  |  |  |
| Clevenger 2024 | US | 44 | 58% | 3-5 | Direct Observation | PA: Accelerometer | Cross-sectional | ✓ |  |  |  |
| Cooper 2010 | United Kingdom | 1010 | 53.3% | 11.0, ±0.4 | Accelerometer/GPS | PA: Accelerometer | Cross-sectional | ✓ |  |  |  |
| Copeland 2016 | US | 388 | 51% | 4.3, ±0.7 | Direct observation | PA:  Accelerometer | Cross-sectional | ✓ |  |  |  |
| Cortinez-O'Ryan 2017 | Chile | 100 | 51% | 9 | Parent report | PA: Pedometer | Quasi-experimental | ✓ |  |  |  |
| daSilva 2024 | Brazil | 204 | 50.5% | 4.5, ±0.79 | Parent/teacher report | PA: Accelerometer | Cross-sectional | ✓ |  |  |  |
| D'Haese 2015 | Belgium | 126 | 45.2% | 9.0, ±2.1 | Parent report | PA and sedentary behaviour: Accelerometer | Quasi-experimental | ✓ | ✓ |  |  |
| Engelen 2015 | Australia | 20 | 45% | 6.3, ±0.6 | Parent report | PA: Accelerometer | Cross-sectional | ✓ |  |  |  |
| Guerrero 2020 | Canada | 1472 | 46.9% | 5-11 and 12-17 | Parent report | PA, screen, and sleep: Parent report | Cross-sectional | ✓ |  | ✓ | ✓ |
| GünayMolu 2022 | Turkey | 442 | 64.3% | 4.7, ±0.7 | Parent report | Sleep: Parent report | Cross-sectional |  |  |  | ✓ |
| Gunter 2012 | US | 136 | 46.3% | 2 -5 | Parent report | PA: Accelerometer | Cross-sectional | ✓ |  |  |  |
| Hager 2017 | US | 160 | 45% | 1.66 | Parent report | PA: Accelerometer | Longitudinal | ✓ |  |  |  |
| Henderson 2015 | US | 389 | 50% | 4.7 | Parent environmental audit report | PA: Accelerometer | Cross-sectional | ✓ |  |  |  |
| Jago 2017 | United Kingdom | 1223 | 54.5% | 9 ±0.41 | Child frequency report | PA and sedentary behaviour: Accelerometer | Cross-sectional | ✓ | ✓ |  |  |
| Jain 2023 | India | 600 | 52% | 8.82, ±3.3 | Parent report | Screen: Parent report | Cross-sectional |  |  | ✓ |  |
| Jiang 2023 | US | 349 | 52% | 3.5, ±0.98 | Direct observation | PA and sedentary behaviour: Accelerometer | Cross-sectional | ✓ | ✓ |  |  |
| Kronaizl 2023 | US | 2320 | 47.8% | 5,9, and 15 | Parent report | PA: Parent report | Longitudinal | ✓ |  |  |  |
| Kwon 2022 | US | 301 | 49.5% | 3-5 | Accelerometer/GPS | PA: Accelerometer | Cross-sectional | ✓ |  |  |  |
| Larouche 2016 | Canada | 42 | 47.8% | 4.7 | Parent report | PA and sedentary behaviour: Accelerometer  Screen: Parent report | Cross-sectional | ✓ | ✓ | ✓ |  |
| Li 2022 | China | 953 | 48.4% | 1,2,3,4, and 5 | Parent report | Screen: Parent report | Longitudinal |  |  | ✓ |  |
| Løndal 2021 | Norway | 42 | 47.6% | 6.5 | Direct observation | Sedentary behaviour and screen: Accelerometry, direct observation | Cross-sectional |  | ✓ |  |  |
| Loucaides 2004 | Cyprus | 256 | Not reported. | 6th grade | Parent and child report | PA: Parent and child report | Cross-sectional | ✓ |  |  |  |
| Lu 2019 | Netherlands | 505 | 49.6% | 3.9, ±0.2 | Parent report | PA and sedentary behaviour: Accelerometer | Cross-sectional | ✓ | ✓ |  |  |
| Luo 2020 | China | 46639 | 49.1% | 10.8, ±3.3 | Parent and child report | Sleep: Parent and child report | Cross-sectional |  |  |  | ✓ |
| Määttä 2019 | Finland | 864 | 48% | 4.7, ±0.9 | Teacher log | Sedentary behaviour: Accelerometer | Cross-sectional |  | ✓ |  |  |
| Manyanga 2019 | Mozambique | 683 | 52.9% | 10.1, ±0.8 | Parent report | PA and sleep: Accelerometer  Screen: Child report | Cross-  sectional | ✓ |  | ✓ | ✓ |
| Marques 2014 | Portugal | 802 | 48% | 10.6, ±0.7 | Parent report | PA and sedentary behaviour: Parent report | Cross-sectional | ✓ | ✓ |  |  |
| Murata 2023 | Japan | 2124 | 49.4% | 2.2 | Parent report | Sleep: Parent report | Cross-sectional |  |  |  | ✓ |
| Nayakarathna 2024 | Canada | 478 | 52.3% | 10 ±0.9 | Parent and child report | PA: Pedometer | Cross-sectional | ✓ |  |  |  |
| Nigg 2021 | Germany | Study 1: 2278 | Study 1: 52.9% | Study 1: 12.5, ±3.3 | Parent report | PA and sedentary behaviour: Accelerometer  Screen: Child report | Cross-sectional | ✓ | ✓ |  |  |
| Nigg 2021 |  | Study 2: 570 | Study 2: 54.7% | Study 2: 5.31, ±0.8; 11.58, ±0.9; 16.54, ±0.8 |  |  | Longitudinal | ✓ |  | ✓ |  |
| Nilsson 2009 | Norway, Denmark, Portugal, Estonia, United Kingdom of Great Britain and Northern Ireland, Sweden | 1327 | 9yr: 49.6% 15 yr: 58.8% | 9.6, ± 0.4; 15.5, ± 0.5 | Child frequency report | PA: Accelerometer  Sedentary behaviour: Child report | Cross-sectional | ✓ | ✓ |  |  |
| Parsons 2018 | US | 359 | 51.5% | 4.4, ± 0.7 | Teacher log | Sleep: Accelerometer | Cross-sectional |  |  |  | ✓ |
| Pate 2024 | US | 951 | 55% | 10-18 | Child, parent, and teacher report | PA: Accelerometer | Longitudinal | ✓ |  |  |  |
| Pearce 2014 | United Kingdom | 427 | 53.9% | 10.7, ±0.5 | Accelerometer/GPS | PA: Accelerometer | Cross-sectional | ✓ |  |  |  |
| Pfledderer 2024 | US | 2897 | 49.40% | 9.4, ± 0.6 | Parent report | PA: Parent report | Cross-sectional | ✓ |  |  |  |
| Sampasa-Kanyinga 2020 | Canada | 10028 | 48.8% | 15.2, ± 1.8 | Child report | PA, screen, and sleep: Child self report | Cross-sectional | ✓ |  | ✓ | ✓ |
| Schaefer 2014 | Canada | 306 | 58% | 13.7, ±1.4 | Child report | PA and sedentary behaviour: Accelerometer | Cross-sectional | ✓ | ✓ |  |  |
| Schenkelberg 2020 | US | 34 | 35.3% | 4.28, ±1.1 | Direct observation | PA: Direct observation | Cross-sectional | ✓ |  |  |  |
| Schmutz 2017 | Switzerland | 394 | 46% | 3.9, ±0.7 | Parent report | PA and sedentary behaviour: Accelerometer | Cross-sectional | ✓ | ✓ |  |  |
| Schoeppe 2014 | Australia | 191 | 62% | 10.6, ±0.9 | Child frequency report | PA: Accelerometer | Cross-sectional | ✓ |  |  |  |
| Silva 2017 | Portugal | 422 | 53.3% | 11.6 | Child frequency report | PA: Accelerometer | Cross-sectional | ✓ |  |  |  |
| Smith 2015 | England | 6558 | 50.8% | 10 | Parent frequency report | Child frequency report | Longitudinal | ✓ |  |  |  |
| Stone 2014 | Canada | 856 | 54.5% | 11, ±0.6 | Parent report | PA and sedentary behaviour: Accelerometer | Cross-sectional | ✓ | ✓ |  |  |
| Tandon 2019 | US | 98 | 57% | 4.6, ±0.4 | Direct observation | PA and sedentary behaviour: Accelerometer | Randomized controlled trial | ✓ | ✓ |  |  |
| Tandon 2015 | US | 97 | 49% | 4.5 | Direct observation | PA and sedentary behaviour: Accelerometer | Cross-sectional | ✓ | ✓ |  |  |
| Tandon 2018 | US | 46 | 36% | 4.5 | Accelerometer/ GPS | PA and sedentary behaviour: Accelerometer | Cross-sectional | ✓ | ✓ |  |  |
| Tey 2007 | Australia | 84 | 57% | 5.1 ± 0.1 | Parent report | Parent report | Cross-sectional | ✓ |  |  |  |
| Tomaz 2019 | South Africa | 55 | 58% | 5 ±0.7 | Direct observation | PA: Direct observation | Cross-sectional | ✓ |  |  |  |
| Triana 2019 | Colombia | 923 | 50.50% | 10.1, ± 0.7 | Parent and child report | PA and sedentary behaviour: Accelerometer | Quasi-experimental | ✓ | ✓ |  |  |
| Vanderloo 2013 | Canada | 31 | 45.2% | 4.1, ±0.9 | Accelerometer and teacher log | PA and sedentary behaviour: Accelerometer | Cross-sectional | ✓ | ✓ |  |  |
| Verbestel 2015 | Belgium, Cyprus, Estonia, Germany, Hungary, Italy, Spain, Sweden | 5882 | 49.40% | 6.1, ±1.8 | Parent report | PA: Accelerometer | Cross-sectional | ✓ |  |  |  |
| Wolfenden 2019 | Australia | 101 | 42% | 4, ±0.7 | Direct observation | PA: Accelerometer | Randomized controlled trial | ✓ |  |  |  |
| Xu 2016 | Australia | 497 | 50% | 2, 3.5, 5 | Parent report | Sleep: Parent report | Longitudinal |  |  |  | ✓ |
| Yamaguchi 2024 | Japan | 187 | 43% | 4.9 ±10.3 | Parent report | PA: Parent report | Cross-sectional | ✓ |  |  |  |
| Zahl-Thanem 2018 | Norway | 1250 | 50.2 % | 6, 8 and 10 | Parent report | PA: Parent report, accelerometer | Longitudinal | ✓ |  |  |  |
| Note. M = mean, SD = standard deviation, AOP = active outdoor play, PA = physical activity | | | | | | | | | | | |
