# Supplementary material for: A systematic review and meta-analyses of the relationships between active outdoor play and 24-hour movement behaviors
Source: J Sport Health Sci. 2025 Dec 29;15:101115. doi: 10.1016/j.jshs.2025.101115 (PMC13053787; doi:10.1016/j.jshs.2025.101115)
Supplement: Supplementary file 4 [file mmc4.docx]

**Appendix D – Complete Risk of Bias Assessments**

| **Cross-sectional** | | | |
| --- | --- | --- | --- |
| **JBI Item** | **Low Risk of Bias (n)** | **Total Studies** | **% Low Risk of Bias** |
| 1: Were the criteria for inclusion in the sample clearly defined? | 40 | 47 | 85 |
| 2: Were the study subjects and the setting described in detail? | 45 | 47 | 96 |
| 3: Was the exposure measured in a valid and reliable way? | 27 | 47 | 57 |
| 4: Were objective, standard criteria used for measurement of the condition? | N/A | N/A | N/A |
| 5: Were confounding factors identified? | 34 | 47 | 72 |
| 6: Were strategies to deal with confounding factors stated? | 37 | 47 | 79 |
| 7: Were the outcomes measured in a valid and reliable way? for All outcomes | 33 | 47 | 70 |
| 8: Was appropriate statistical analysis used? | 46 | 47 | 98 |
| **Longitudinal (Cohort)** | | | |
| 1: Were the two groups similar and recruited from the same population? | 5 | 7 | 71 |
| 2: Were the exposures measured similarly to assign people to both exposed and unexposed groups? | 6 | 7 | 86 |
| 3: Was the exposure measured in a valid and reliable way? | 1 | 7 | 14 |
| 4: Were confounding factors identified? | 7 | 7 | 100 |
| 5: Were strategies to deal with confounding factors stated? | 7 | 7 | 100 |
| 6: Were the groups/participants free of the outcome at the start of the study (or at the moment of exposure)? | 0 | 7 | 0 |
| 7: Were the outcomes measured in a valid and reliable way? for All outcomes | 4 | 7 | 57 |
| 8: Was the follow up time reported and sufficient to be long enough for outcomes to occur? | 7 | 7 | 100 |
| 9: Was follow up complete, and if not, were the reasons to loss to follow up described and explored? | 2 | 7 | 29 |
| 10: Were strategies to address incomplete follow up utilized? | 5 | 7 | 71 |
| 11: Was appropriate statistical analysis used? | 7 | 7 | 100 |
| **Quasi-Experimental** | | | |
| 1: Is it clear in the study what is the cause and what is the effect (i.e. there is no confusion about which variable comes first)? | 4 | 4 | 100 |
| 2: Was there a control group? | 4 | 4 | 100 |
| 3: Were participants included in any comparisons similar? | 2 | 4 | 50 |
| 4: Were the participants included in any comparisons receiving similar treatment/care, other than the exposure or intervention of interest? | 3 | 4 | 75 |
| 5: Were there multiple measurements of the outcome, both pre and post the intervention/exposure? for All outcomes | 5 | 4 | 100 |
| 6: Were the outcomes of participants included in any comparisons measured in the same way? for All outcomes | 5 | 4 | 100 |
| 7: Were outcomes measured in a reliable way? for All outcomes | 3 | 4 | 75 |
| 8: Was follow-up complete and if not, were differences between groups in terms of their follow-up adequately described and analyzed? | 1 | 4 | 25 |
| 9: Was appropriate statistical analysis used? | 2 | 4 | 50 |
| **Randomized Controlled Trial** | | | |
| 1: Was true randomization used for assignment of participants to treatment groups? | 3 | 3 | 100 |
| 2: Was allocation to treatment groups concealed? | 0 | 3 | 0 |
| 3: Were treatment groups similar at the baseline? | 3 | 3 | 100 |
| 4: Were participants blind to treatment assignment? | 0 | 3 | 0 |
| 5: Were those delivering the treatment blind to treatment assignment? | 0 | 3 | 0 |
| 6: Were treatment groups treated identically other than the intervention of interest? | 3 | 3 | 100 |
| 7: Were outcome assessors blind to treatment assignment? | 0 | 3 | 0 |
| 8: Were outcomes measured in the same way for treatment groups? for All outcomes | 3 | 3 | 100 |
| 9: Were outcomes measured in a reliable way for all outcomes | 3 | 3 | 100 |
| 10: Was follow-up complete and if not, were differences between groups in terms of their follow-up adequately described and analyzed? | 2 | 3 | 67 |
| 11: Were participants analyzed in the groups to which they were randomized? | 3 | 3 | 100 |
| 12: Was appropriate statistical analysis used? | 3 | 3 | 100 |
| 13: Was the trial design appropriate and any deviations from the standard RCT design (individual randomization, parallel groups) accounted for in the conduct and analysis of the trial? | 3 | 3 | 100 |

Note. N/A = Not Applicable
