# Supplementary material for: A systematic review and meta-analyses of the relationships between active outdoor play and 24-hour movement behaviors
Source: J Sport Health Sci. 2025 Dec 29;15:101115. doi: 10.1016/j.jshs.2025.101115 (PMC13053787; doi:10.1016/j.jshs.2025.101115)
Supplement: Supplementary file 5 [file mmc5.docx]

| **Appendix E – GRADE Assessment by Study Design and Outcome** | | | | | | | | |
| --- | --- | --- | --- | --- | --- | --- | --- | --- |
| **Outcome** | **Study Design** | **Risk of Bias Concerns** | **Inconsistency Concerns** | **Indirectness Concerns** | **Imprecision Concerns** | **Publication Bias Concerns** | **Final Certainty Rating** | **Justification Summary** |
| Physical activity | Cross-sectional | **No** - Most studies were low risk of bias overall. While many used unvalidated or potentially unreliable measures of outdoor play this reflects the current state of the field and does not warrant downgrading. | **No** - Approximately 75% of studies showed favourable associations, with no opposing effects. | **No** - All studies directly measured outdoor play and movement behaviours, matching the research question. Although limited to children and youth, this group is a key intervention target, making findings relevant to the broader aim. | **No** - Large total sample size (N = 39,526) and consistency in direction reduce concerns. | **No** - Range of findings across many studies reduces concern for selective publication. | **LOW** | Although most studies used unvalidated or unreliable measures for outdoor play, this is a common limitation in the field and was not considered serious enough to warrant downgrading. Valid and reliable measures were generally used to measure physical activity and the sample size was large. Other domains were not serious concerns. Therefore, the rating remains at low |
| Physical activity | Longitudinal | **No** - Most studies were low risk of bias with the exception of not using validated/reliable measures for outdoor play. Given that this is a limitation of the field, this was not deemed a serious risk of bias. | **No** - Results were mostly consistent: 4 of 6 longitudinal studies showed a favourable link between outdoor play and physical activity. The two null studies examined long-term effects into adulthood, while others focused on shorter childhood/adolescence periods. Differences in follow-up time likely explain the variation, so inconsistency is not serious. | **No** - All studies directly measured outdoor play and movement behaviours, matching the research question. Although limited to children and youth, this group is a key intervention target, making findings relevant to the broader aim. | **No** - The six longitudinal studies had a large total sample (N = 14,087), with 4 showing favourable associations. Consistent findings and adequate sample size support rating imprecision as not serious. | **No** - Both favourable and null findings were identified across studies, reducing concern for selective publication. No evidence suggesting publication bias was detected; therefore, this domain was rated as not serious. | **LOW** | While many studies relied on unvalidated or less reliable measures for the exposure variable, this is a common issue in this field this was not deemed severe enough to justify downgrading. No other domains raised serious concerns, so the rating stays at low. |
| Physical Activity | Intervention – Non-Randomized trials | **No** - Both studies had low risk of bias across most domains, with some concern for follow-up; this was not likely to substantially bias results. | **No** - Both studies showed similar favourable effects of the play streets intervention on physical activity. | **No** - Populations, interventions, and outcomes aligned with the review question. | **Yes** - Only two studies were available, with relatively small sample sizes and no pooled effect estimate; thus, uncertainty around the precision of the effect remains. | **No** - A comprehensive search was conducted, and both studies were identified regardless of direction of findings. | **VERY LOW** | Rated very low due to study design (non-randomized) and imprecision (only two small studies with limited follow-up data). Findings were consistent and directly aligned with the research question. |
| Physical Activity | Intervention –Randomized trials | **Yes** - Both trials lacked blinding and allocation concealment, increasing the risk of performance and detection bias. | **No** - Both studies found null effects, so the findings are consistent in direction. | **No** - Populations, interventions, and outcomes aligned with the review question. | **Yes -** Very small sample sizes (n = 98 and n = 32) with null results increase uncertainty in the true effect estimate. | **No -** No indication of missing studies, and both null findings were published. | **VERY LOW** | Rated very low due to risk of bias (lack of blinding and allocation concealment) and imprecision (small sample sizes with null findings). Results were consistent and directly aligned with the research question. |
| Sedentary Behaviour | Cross-sectional | **No** - Three studies did not identify or control for confounding, and some studies used unreliable exposure measures which is a field-wide limitation; however, these issues were not deemed serious enough to warrant downgrading. | **No** - Most studies (12/17) showed favourable associations, one showed mixed results, and four showed null findings. The direction is generally consistent with no contradictory effects. | **No** - All studies directly measured outdoor play and movement behaviours, matching the research question. Although limited to children and youth, this group is a key intervention target, making findings relevant to the broader aim. | **No** - The total sample size is substantial (N = 16,240), reducing concerns about imprecision. | **No** - The presence of null and mixed results reduces concerns about selective publication. | **LOW** | The evidence starts at low certainty given the cross-sectional design. Risk of bias, inconsistency, indirectness, imprecision, and publication bias were not serious concerns. |
| Sedentary Behaviour | Intervention – Non-Randomized trials | **No** - Both studies were generally low risk of bias. | **No** - Both studies found favourable effects (reduced sedentary behaviour). | **No** - Populations, interventions (play streets), and outcomes (sedentary behaviour) directly align with the review question. | **Yes** - Only two studies with small sample sizes (n = 98 and n = 126). | **No** - Both studies were published regardless of findings; no obvious reason to suspect missing evidence. | **VERY LOW** | Rated very low due to study design (non-randomized) and imprecision (only two small studies with subgroup-specific findings). Risk of bias was low, and findings were consistent and directly aligned with the review question. |
| Sedentary Behaviour | Intervention –Randomized trials | **Yes** - The study was low risk of bias in most domains, but lack of blinding (especially if outcome was self-reported) introduces potential for performance or detection bias. | **Yes** - Downgrading because there is only one study and thus there is an inability to assess inconsistency | **No** - The population, intervention, and outcome aligned with your review question. | **Yes** - Small sample size (n = 98), null finding suggests uncertainty in the effect estimate. | **No** - No evidence of selective reporting; the null result was published. | **VERY LOW** | Rated very low due to risk of bias (lack of blinding), imprecision (small sample size and null result), and inability to assess consistency with only one study. |
| Screen time | Cross-sectional | **No** - Most studies were low risk of bias with the exception of not using validated/reliable measures for outdoor play. Given that this is a limitation of the field, this was not deemed a serious risk of bias. | **No** - 3 of 5 studies showed favourable associations, 2 showed no association, but no studies reported adverse or opposite effects. | **No** - All studies directly measured outdoor play and movement behaviours, matching the research question. Although limited to children and youth, this group is a key intervention target, making findings relevant to the broader aim. | **No** - Total sample size is substantial (N=13,222), which reduces concern for imprecision despite the mixed findings. | **No** - Variety of findings reduces likelihood of publication bias. | **LOW** | The evidence starts at low certainty given the cross-sectional design. Although most studies used unvalidated or unreliable measures to assess outdoor play, this is a common limitation in the field and was not considered serious enough to warrant downgrading. Other domains were not serious concerns. Therefore, the rating remains as low |
| Screen time | Longitudinal | **Yes** - Both studies had high risk of bias due to incomplete follow-up and unclear reasons for loss to follow-up, which may introduce attrition bias. Measurement limitations are common and less concerning, but combined with follow-up issues, this warrants downgrading | **Yes** - The findings are mixed and somewhat contradictory | **No** - All studies directly measured outdoor play and movement behaviours, matching the research question. Although limited to children and youth, this group is a key intervention target, making findings relevant to the broader aim. | **No** - Combined sample size is fairly large (n = 2848 + 594), reducing concerns about imprecision. | **No -** No evidence of selective publication. | **VERY LOW** | The evidence starts at low certainty due to the observational longitudinal design. Downgrading was made for serious risk of bias related to incomplete follow-up and unclear reasons for attrition, and for inconsistency given mixed and conflicting results across studies. Measurement limitations, though present, were considered common in the field and did not result in additional downgrading. Other domains were not serious concerns. |
| Sleep | Cross-sectional | **Yes** - Five of eight studies used invalid or unreliable measures for sleep, and most did not use valid or reliable measures for outdoor play (although a common field limitation so not necessarily a serious concern). One study did not identify or control for confounding factors, increasing risk of bias. | **Yes** - Mixed findings across all studies and variability in outcomes measured (i.e., quality vs. quantity) | **No -** All studies directly measured outdoor play and movement behaviours, matching the research question. Although limited to children and youth, this group is a key intervention target, making findings relevant to the broader aim. | **No** - Large total sample size (N=61,320) reduces concern about imprecision despite inconsistent findings. | **No** - Range of findings across many studies reduces suspicion of selective publication. | **VERY LOW** | The evidence starts at low certainty given the cross-sectional design. Downgrades were made for risk of bias due to widespread use of unreliable or invalid measures for sleep and outdoor play, and lack of confounding control in one study. Inconsistency was also downgraded due to mixed findings across outcomes and measures. Other domains were not serious concerns. |
