# Supplementary material for: A systematic review and meta-analyses of the relationships between active outdoor play and 24-hour movement behaviors
Source: J Sport Health Sci. 2025 Dec 29;15:101115. doi: 10.1016/j.jshs.2025.101115 (PMC13053787; doi:10.1016/j.jshs.2025.101115)
Supplement: Supplementary file 6 [file mmc6.docx]

|  | | **Appendix F – Characteristics of studies included in the meta-analyses** | | | | | | | |
| --- | --- | --- | --- | --- | --- | --- | --- | --- | --- |
| **Author, year** | **Age**  **(M, SD)** | | **Exposure Variable** | **Outcome Measurement** | **Sample size** | **SDy** | **Unstandardized Beta-Coefficient/Odds Ratio** | **Pearson r (calculated)** | **Pearson r (reported in paper)** |
| **Moderate- to Vigorous-intensity Physical Activity (MVPA)** | | | | | | | | | |
| Aggio 2017 | 7.2, ±0.3 | | Independent outdoor play (yes/no) | Accelerometer | 6,442 | 22.3 | 2.210 | 0.050 | N/A |
| Chen 2020a^1^ | 4.7, ±0.8 | | Outdoor time Q2 vs. Q1 | Accelerometer | 369 | 1.20 | -3.400 | -0.815 | N/A |
| Chen 2020b^1^ | 4.7, ±0.8 | | Outdoor time Q3 vs. Q1 | Accelerometer | 369 | 1.20 | 4.600 | 0.760 | N/A |
| Chen 2020c^1^ | 4.7, ±0.8 | | Outdoor time Q4 vs. Q1 | Accelerometer | 369 | 1.20 | 11.500 | 0.714 | N/A |
| Copeland 2016a | 4.3, ±0.7 | | Outdoor time > 60 minutes at childcare centre | Accelerometer | 365 | 0.08 | 0.500 | 0.725 | N/A |
| Copeland 2016b | 4.3, ±0.7 | | Outdoor time > 60 minutes over the whole day | Accelerometer | 365 | 0.06 | 0.500 | 0.717 | N/A |
| Loucaides 2004a | Not reported. | | Hours per day playing outside (Summer) | Parent and child report | 256 | N/A | 0.286 | N/A | 0.248 |
| Loucaides 2004b | Not reported. | | Hours per day playing outside (Winter) | Parent and child report | 256 | N/A | 0.271 | N/A | 0.248 |
| Pearce 2014a | 10.7, ±0.5 | | Outdoor time alone (boys) | Accelerometer | 197 | 3.40 | -0.410 | -0.06 | N/A |
| Pearce 2014b | 10.7, ±0.5 | | Outdoor time with friend (boys) | Accelerometer | 197 | 8.10 | 17.530 | 0.932 | N/A |
| Pearce 2014c | 10.7, ±0.5 | | Outdoor time with sibling (boys) | Accelerometer | 197 | 3.70 | 16.950 | 0.742 | N/A |
| Pearce 2014d | 10.7, ±0.5 | | Outdoor time with parent (boys) | Accelerometer | 197 | 4.80 | 9.000 | 0.939 | N/A |
| Pearce 2014e | 10.7, ±0.5 | | Outdoor time with other grown up (boys) | Accelerometer | 197 | 5.10 | 8.540 | 0.838 | N/A |
| Pearce 2014f | 10.7, ±0.5 | | Outdoor time alone (girls) | Accelerometer | 230 | 3.70 | 7.270 | 0.984 | N/A |
| Pearce 2014g | 10.7, ±0.5 | | Outdoor time with friend (girls) | Accelerometer | 230 | 6 | 17.350 | 0.809 | N/A |
| Pearce 2014h | 10.7, ±0.5 | | Outdoor time with sibling (girls) | Accelerometer | 230 | 3.30 | 21.210 | 0.723 | N/A |
| Pearce 2014i | 10.7, ±0.5 | | Outdoor time with parent (girls) | Accelerometer | 230 | 3.80 | 5.550 | 0.73 | N/A |
| Pearce 2014j | 10.7, ±0.5 | | Outdoor time with other grown up (girls) | Accelerometer | 230 | 5.10 | 12.760 | 0.855 | N/A |
| Schmutz 2017 | 3.9, ±0.7 | | Time Outdoors | Accelerometer | 394 | 30 | 1.500 | 0.025 | N/A |
| Schoeppe 2014 | 10.6, ±0.9 | | Time outdoors (categories) | Accelerometer | 191 | 45.90 | 3.960 | 0.043 | N/A |
| Verbestel 2015a ^2^ | 6.1, ±1.8 | | OPRQ Derived outdoor play (mins/day) | Accelerometer | 5,982 | 3.07 | 0.005 | 0 | N/A |
| Verbestel 2015b ^3^ | 6.1, ±1.8 | | OPC Derived outdoor play (hrs/per min) | Accelerometer | 5,982 | 3.07 | 0.050 | 0.003 | N/A |
|  | | **Total Physical Activity (of any intensity)** | | | | | | | |
| Verbestel 2015a ^2^ | 6.1, ±1.8 | | OPRQ Derived outdoor play (mins/day) | PA counts per minute | 5,982 | 165.49 | 0.290 | 0.0009 | N/A |
| Verbestel 2015b ^3^ | 6.1, ±1.8 | | OPC Derived outdoor play (hrs and mins) | PA counts per minute | 5,982 | 165.49 | 2.930 | 0.0089 | N/A |
| Schoeppe 2014 | 10.6, ±0.9 | | 3 days or more of outdoor play | Total PA | 191 | 95.30 | 32.430 | 0.1699 | N/A |
| Kwon 2022a | 3-5 | | Time outdoors | Daily overall PA (boys) | 152 | 595 | 296 | 0.2484 | N/A |
| Kwon 2022b | 3-5 | | Time outdoors | Daily overall PA (girls) | 149 | 432 | 540 | 0.6248 | N/A |
| Schmutz 2017 | 3.9, ±0.7 | | Time outdoors | TPA counts per mins | 476 | 150 | 12.500 | 0.0416 | N/A |
| Marques 2014a | 10.6, ±0.7 | | Frequency of outdoor play (boys) | Overall PA | 416 | 27.90 | 0.056 | 0.001 | N/A |
| Marques 2014b | 10.6, ±0.7 | | Frequency of outdoor play (girls) | Overall PA | 386 | 21.40 | -0.500 | -0.0117 | N/A |
|  | | **Sedentary Behaviour** | | | | | | | |
| Aggio 2017 | 7.2, ±0.3 | | Independent outdoor play (yes/no) | Accelerometer | 6,442 | 67 | -4.910 | -0.0366 | N/A |
| Lu 2019 | 3.9, ±0.2 | | Time in outdoor play per week | Accelerometer | 505 |  | -1 | -0.12 | N/A |
| Maatta 2019 | 4.7, ±0.9 | | Times outdoors per day | Accelerometer | 778 | 5.10 | -0.259 | -0.0254 | N/A |
| Nigg 2021a | **DC | | Medium outdoor play (1hr/day) | Accelerometer | 2,278 | 122.73 | -9.750 | -0.0397 | N/A |
| Nigg 2021b | **DC | | High outdoor play (2hr/day) | Accelerometer | 2,278 | 122.73 | -17.780 | -0.0724 | N/A |
| Schmutz 2017 | 3.9, ±0.7 | | Time outdoors | Accelerometer | 394 | 48 | -4.700 | -0.0489 | N/A |
|  | | **Screen Time** | | | | | | | |
| Jain 2023 | 8.82, ±3.3 | | 2 hours or more of outdoor play per day | Screen viewing less than 2 hours per day | 600 | N/A | 5.17^4^ | -0.4070 | N/A |
| Larouche 2016 | 4.7 | | Time outdoors | Total screen time | 293 | 2.62 | -0.2 | -0.0381 | N/A |
| Sampasa-Kanyinga 2019a | 15.2, ± 1.8 | | Outdoor play frequency (1-2 days vs. 0 days) | Less than or equal to 2  hours per day of screen time | 10,028 | N/A | 1.18^4^ | -0.0452^5^ | N/A |
| Sampasa-Kanyinga 2019b | 15.2, ± 1.8 | | Outdoor play frequency (3-4 days vs. 0 days) | Less than or equal to 2  hours per day of screen time | 10,028 | N/A | 1.69^4^ | -0.1431^5^ | N/A |
| Sampasa-Kanyinga 2019c | 15.2, ± 1.8 | | Outdoor play frequency (All 5 days vs. 0 days) | Less than or equal to 2  hours per day of screen time | 10,028 | N/A | 3.02^4^ | -0.2903^5^ | N/A |
|  | | **Sleep** | | | | | | | |
| Parsons 2018 | 4.4, ± 0.7 | | Total active time outdoors (mins) | Accelerometer | 359 | 0.97 | 0.015 | 0.0077 | N/A |
| Xu 2016a | 2, 3.5, 5 | | Outdoor playtime (hrs/day) | Parent Report | 497 | 1.1 | -0.020 | -0.0091 | N/A |
| Xu 2016b | 2, 3.5, 5 | | Outdoor playtime (boys) | Parent Report | 415 | 0.97 | 0.080 | 0.0412 | N/A |
| Xu 2016c | 2, 3.5, 5 | | Outdoor playtime (girls) | Parent Report | 369 | 0.81 | -0.010 | -0.0062 | N/A |
|  | | Notes.  ^1^Q1 = *<*138min, Q2 = 138min ≤ to *<*187.5min, Q3 = 187.5 min ≤ to *<*234min and Q4 ≥ 234min  ^2^OPRQ = Outdoor Playtime Recall Questions, assesses time children played outdoors during the past month  ^3^OPC = Outdoor Playtime Checklist, assesses daily time usually spent in outdoor play in two different locations: i) yard or street around the house ii) park, playground or outdoor recreation  ^4^Odds ratio used for correlation coefficient calculation  ^5^Effect size reverse-coded  SDy = standard deviation y-variable; N/A = not applicable; PA = physical activity | | | | | | | |
